# Moraines in the Austrian Alps record repeated phases of glacier stabilization through the Late Glacial and the Early Holocene

**DOI:** 10.1038/s41598-022-12477-x

**Published:** 2022-06-13

**Authors:** Sandra M. Braumann, Joerg M. Schaefer, Stephanie Neuhuber, Markus Fiebig

**Affiliations:** 1 Institute of Applied Geology, University of Natural Resources and Life Sciences (BOKU), Peter Jordan-Straße 82, 1190 Vienna, Austria; 2 Division of Geochemistry, Lamont-Doherty Earth Observatory of Columbia University, Palisades, NY 10964 USA

**Keywords:** Climate sciences, Climate change, Cryospheric science, Palaeoclimate, Geochemistry, Stratigraphy, Geochemistry, Geology, Geomorphology, Sedimentology

## Abstract

Climate is currently warming due to anthropogenic impact on the Earth’s atmosphere. To better understand the processes and feedbacks within the climate system that underlie this accelerating warming trend, it is useful to examine past periods of abrupt climate change that were driven by natural forcings. Glaciers provide an excellent natural laboratory for reconstructing the climate of the past as they respond sensitively to climate oscillations. Therefore, we study glacier systems and their behavior during the transition from colder to warmer climate phases, focusing on the period between 15 and 10 ka. Using a combination of geomorphological mapping and beryllium-10 surface exposure dating, we reconstruct ice extents in two glaciated valleys of the Silvretta Massif in the Austrian Alps. The mountain glacier record shows that general deglaciation after the Last Glacial Maximum (LGM) was repeatedly interrupted by glacier stabilization or readvance, perhaps during the Oldest Dryas to Bølling transition (landform age: 14.4 ± 1.0 ka) and certainly during the Younger Dryas (YD; 12.9–11.7 ka) and the Early Holocene (EH; 12–10 ka). The oldest landform age indicates a lateral ice margin that postdates the ‘Gschnitz’ stadial (ca. 17–16 ka) and predates the YD. It shows that local inner-alpine glaciers were more extensive until the onset of the Bølling warm phase (ca. 14.6 ka), or possibly even into the Bølling than during the subsequent YD. The second age group, ca. 80 m below the (pre-)Bølling ice margin, indicates glacier extents during the YD cold phase and captures the spatial and temporal fine structure of glacier retreat during this period. The ice surface lowered approximately 50–60 m through the YD, which is indicative of milder climate conditions at the end of the YD compared to its beginning. Finally, the third age group falls into a period of more substantial warming, the YD–EH transition, and shows discontinuous glacier retreat during the glacial to interglacial transition. The new geochronologies synthesized with pre-existing moraine records from the Silvretta Massif evidence multiple cold phases that punctuated the general post-LGM warming trend and illustrate the sensitive response of Silvretta glaciers to abrupt climate oscillations in the past.

## Introduction

Mountain glaciers are highly sensitive to climate variations, most importantly to changes in summer temperatures and to a lesser extent to changes in precipitation^1,2^. This sensitivity is evident in the accelerating deglaciation of alpine regions caused by rapid warming due to increasing greenhouse gas emissions in recent decades^3^. Deglaciation affects mountain regions in various ways; ice retreat alters the hydrological regime in these areas and downstream, impacts ecosystems, and increases the frequency of natural hazards^4^. Improving our understanding of interactions between the climate system and the cryosphere in the past helps project the magnitude and impact of environmental change in the future.

Glaciers and their shaping of many parts of the Earth’s surface in the past^5,6^ enable us to explore the climate system and how it operates with and without anthropogenic impact. Detailed geomorphological mapping and direct dating of former ice margins allow us to reconstruct glaciers across time and space and to draw conclusions about climate conditions that drove glacier advance or retreat. Here, we present ice-margin reconstructions of two glaciated valleys in the Austrian Alps, Jamtal and Fimbatal, covering the Late Glacial (LG) and the Early Holocene (EH)—a period when the climate was transitioning from a glacial to an interglacial mode.

Late Glacial moraines in the European Alps, especially those deposited during the Younger Dryas (YD, ca. 12.9–11.7 ka) termed ‘Egesen’ moraines, are subject to numerous geochronological studies that provide valuable insights into this last phase of prolonged cooling before Holocene warming^7^. Other periods of climate transition before and after the YD remain more controversial in terms of glacier extents and ice dynamics, for instance, the Bølling–Allerød period (B–A, ca. 14.6–12.9 ka) and the EH (ca. 11.7–9 ka). Although glaciers were presumably much more extensive at the beginning of the B–A compared to the beginning of the EH, they were driven by a similar climatic pattern during both periods: an abrupt temperature increase which led to rapid deglaciation. For the period between ca. 12 and 10 ka, moraine chronologies from different places in the Alps, including data presented in this study, have shown that ice retreat and therefore warming was not linear but interrupted by centennial-scale cooling^8–11^. For the (pre-)Bølling period, it is still unclear whether ice retreat was steady and of such magnitude that most glaciers in the European Alps retreated to the highest cirques or disappeared altogether^7^. The objective of this study is to contribute to these less resolved periods and to view climate transitions from glacial to interglacial conditions through the lens of glacial geomorphology. Therefore, we mapped moraine sequences at two alpine valleys, the Jamtal and the Fimbatal, and applied ^10^Be surface exposure dating to selected landforms to produce direct spatial and temporal information of former ice extents in the Silvretta Massif.

**Figure 1:**
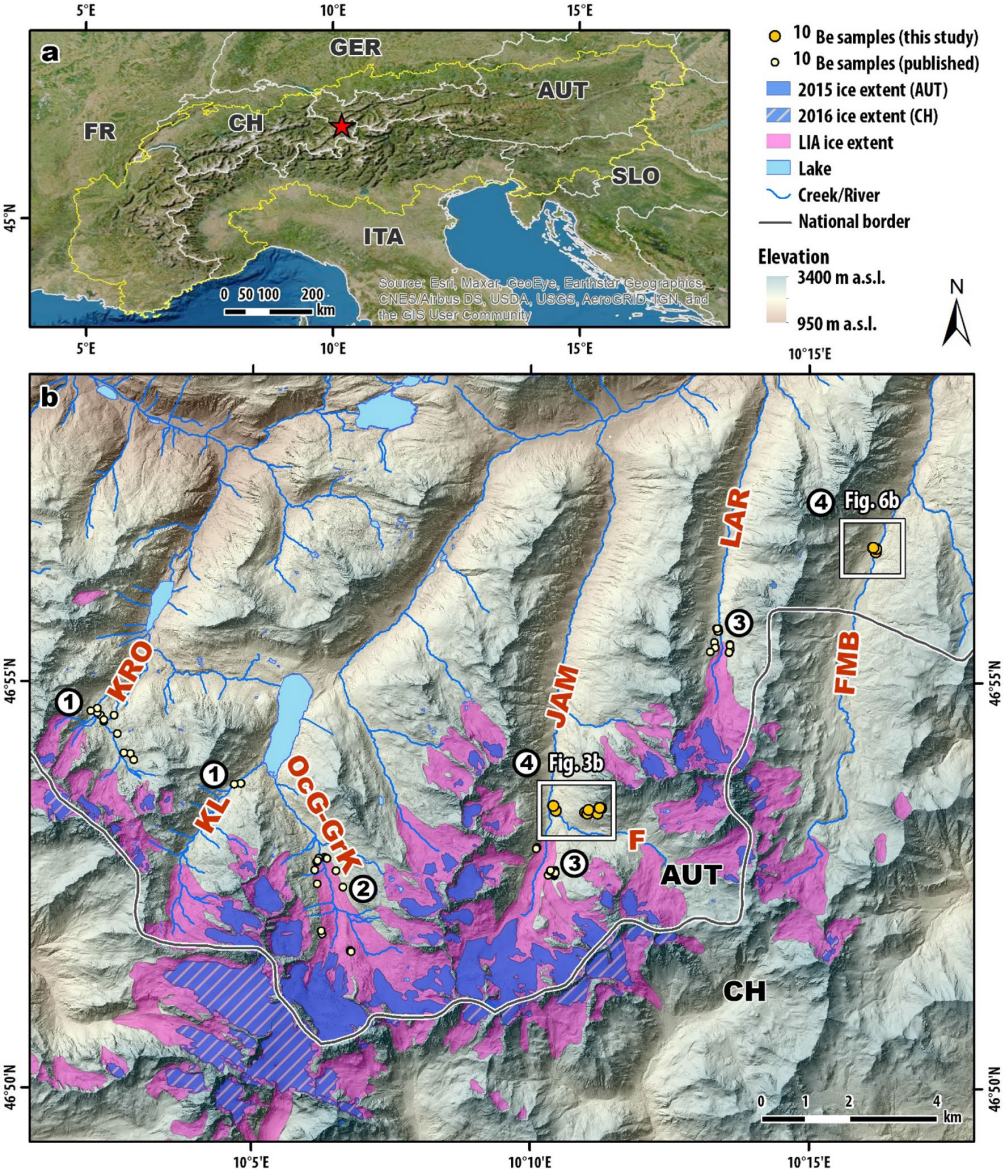
Location of study sites. **(a)** Overview of the European Alps (yellow line); red star symbol marks Silvretta region, a glaciated area at the transition zone between the Western and the Eastern Alps. Sattelite image provided by © 2020 Esri and its licensors (https://services.arcgisonline.com/ArcGIS/rest/services/World_Imagery/MapServer). (**b**) Investigated valleys in the north-facing part of the Silvretta region. Ice extents during the Little Ice Age (LIA) are depicted in pink; modern glacier extents are indicated with blue signature (glacier extents of 2016 for Switzerland CH; glacier extents 2015 for Austria AUT)^15–17^. DEMs provided by ©swisstopo (https://www.swisstopo.admin.ch/en/geodata/height/alti3d.html), Land Tirol (www.tirol.gv.at/als; CC BY 4.0), and Land Vorarlberg (https://vogis.cnv.at/atlas/init.aspx; CC BY 4.0). Valleys with previously published ^10^Be moraine records from West to East: Kromertal (KRO) and Klostertal (KL) #1: Moran, et al.﻿^18^, Ochsental (OcG-GrK) #2: Braumann, et al.﻿^19^, Jamtal (JAM) with tributary valley Futschöltal (F) and Laraintal (LAR) #3: Braumann, et al.^9^ and #4: this study, FMB—Fimbatal (#4: this study). Maps were created using ArcGIS v10.7.

**Figure 2:**
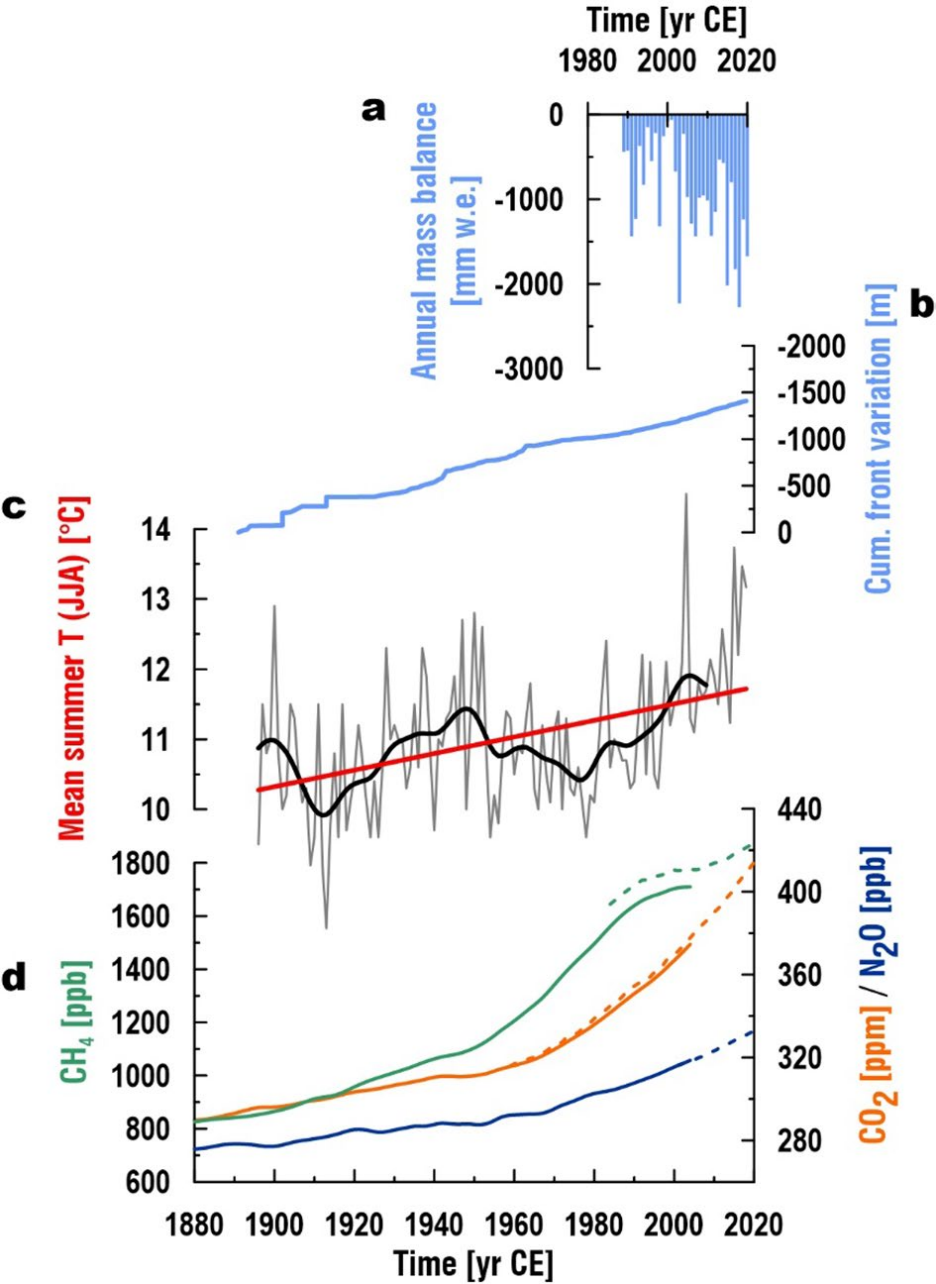
Response of Jamtal glacier to greenhouse gas emissions and rising temperatures over the past decades. **(a)** Mass balance record of Jamtal glacier (1989–2020 CE) with negative values ranging between − 62 mm water equivalent (w.e.) (2000 CE) and − 2276 mm water equivalent (2018 CE)^﻿20^. **(b)** Front variation of Jamtal glacier (1891–2019) showing retreat of ca. 1.5 km since 1891 CE^20^. **(c)** Mean summer temperature (grey line) at meteorological station Galtür (station number: 101949; 1587 m a.s.l.) overlain with 20 years low pass filter (black line)^21,22^﻿. **(d)** Mean annual atmospheric greenhouse gas concentrations (1880–2004) derived from Law Dome ice core (solid lines)^23^. Globally averaged marine surface annual data of CH_4_ (1984–2020 CE; green dashed line), CO_2_ (1959–2020; orange dashed line), and N_2_O (2001–2020; blue dashed line) were provided by NOAA Global Monitoring Laboratory^24^.

**Figure 3:**
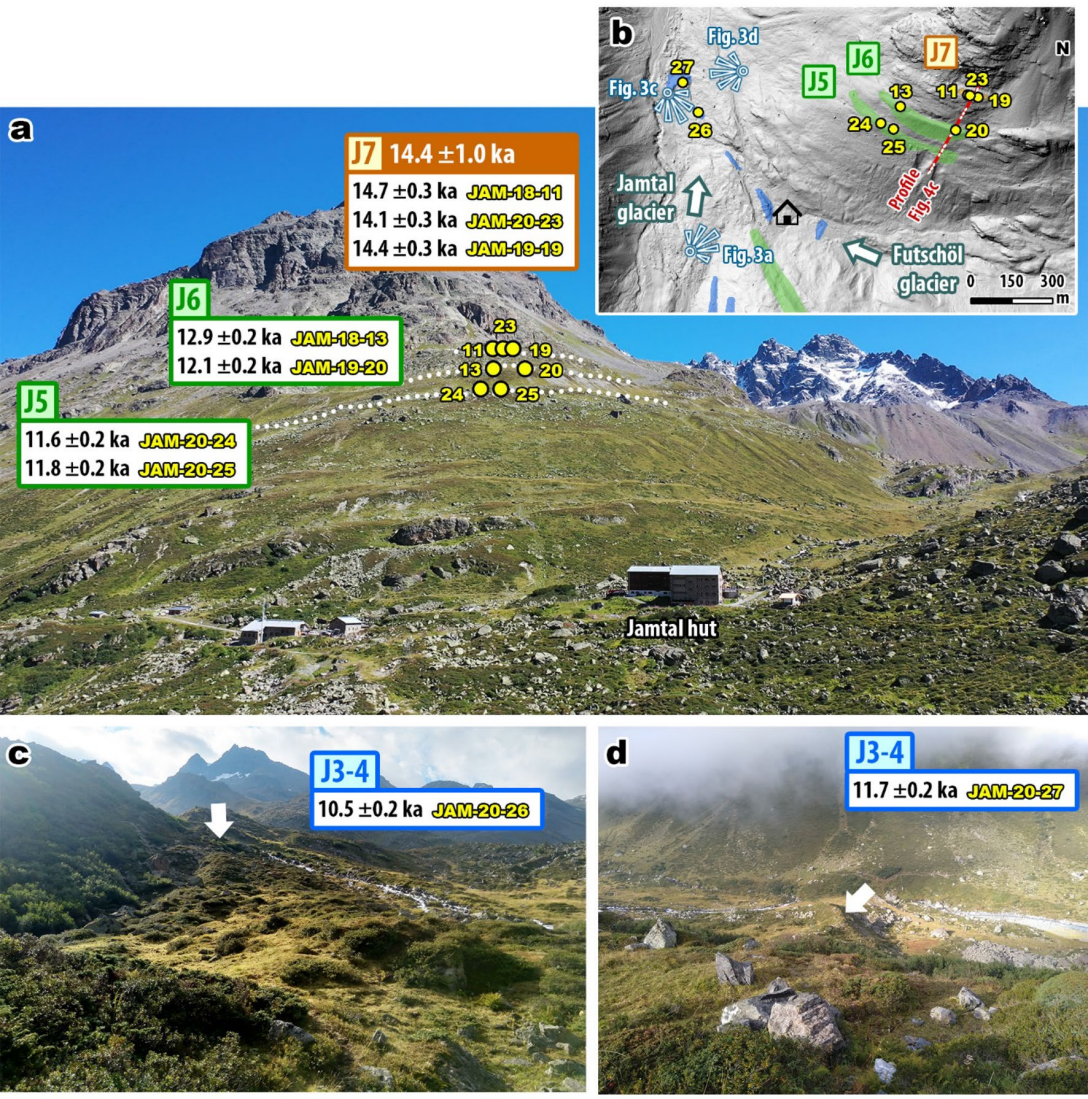
Photographs and map of sampled valley sections at Jamtal. **(a)** Moraine sequence (dotted lines) evidence confluence of tributary (Futschöl) glacier with the main (Jamtal) glacier during the LG. The uppermost landform J7 indicates an ice margin position during (pre-)Bølling times. Moraines J6 and J5 were deposited during the YD and suggest a lowering of the ice surface of ca. 55 m from the early YD to its end. **(b)** Map showing sample and landform locations at Jamtal (DEM provided by Land Tirol—www.tirol.gv.at/als, CC BY 4.0; map was created using ArcGIS v10.7). **(c)** Lateral moraine set deposited during the EH. The inner of two ridges (arrow) features boulder age JAM-20-26 and is (due to its position) several centuries younger compared to **(d)** sample JAM-20-27 taken from the terminal section of J3–4. The statistically identical ages of J5 and JAM-20-27 suggest that the valley flank displayed in (**a**) deglaciated within centuries during the YD–EH transition.

**Figure 4:**
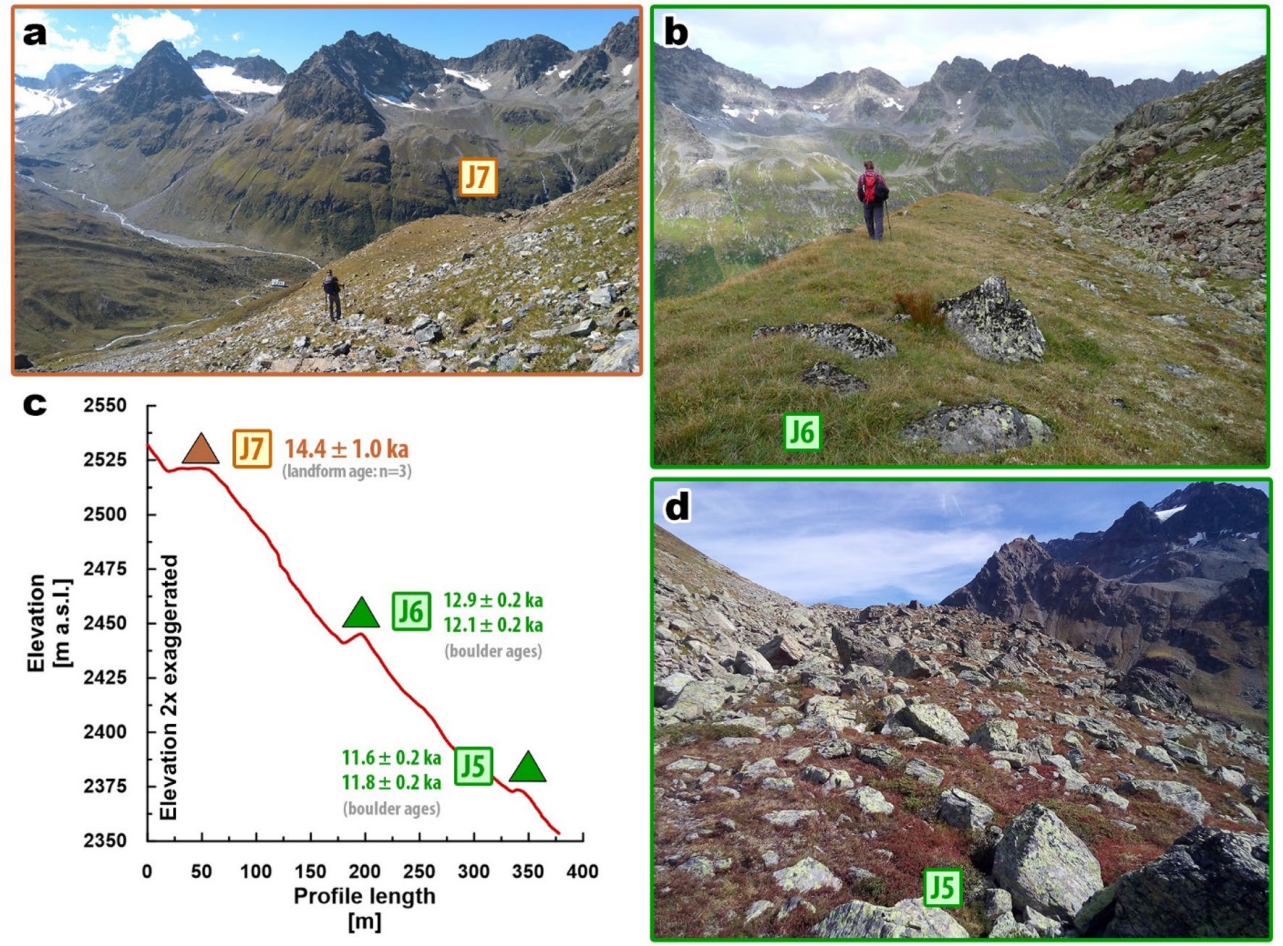
Closeups of dated moraines **(a)** J7, **(b)** J6 and **(d)** J5 highlight different geomorphological characteristics of the three landforms. **(c)** Profile along LG valley flank (for location, see Fig. 3b) indicating ice surface positions at different times during the LG.

**Figure 5:**
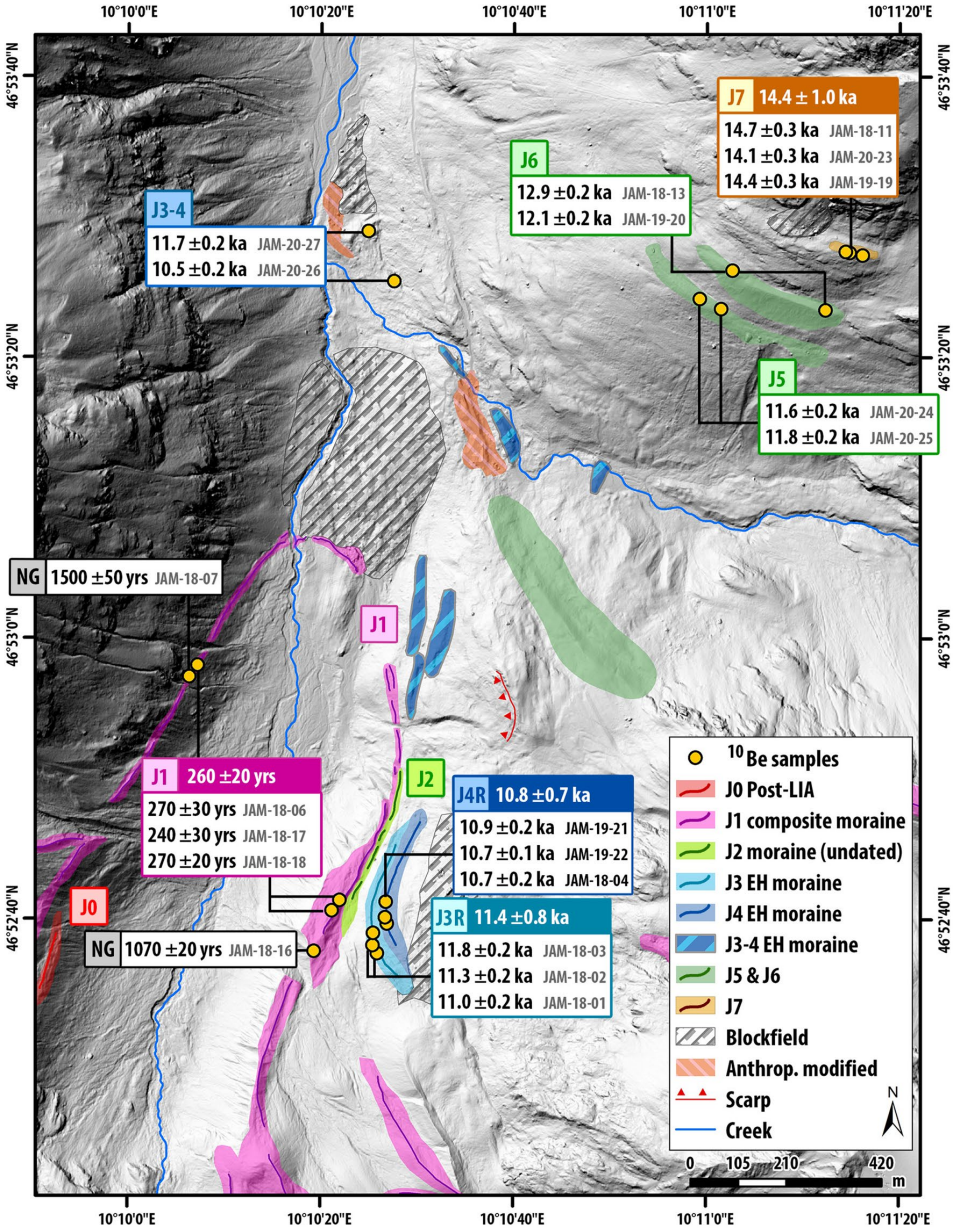
Late Glacial and Holocene moraine chronology of Jamtal. Boulder ages depicted in the lower half of the map (i.e., Holocene ages except samples JAM-20-26 and JAM-20-27) are published in Braumann, et al.^9^ New ages presented in this study (top half of the map) complement the Holocene time scale and indicate ice margin positions during the Oldest Dryas–Bølling transition (J7 in brown) and during the YD (J5 and J6 in green). DEM provided by Land Tirol—www.tirol.gv.at/als, CC BY 4.0. The map was created using ArcGIS v10.7.

The Silvretta Massif is in the westernmost part of the Eastern European Alps (Fig. 1a). The mountain range belongs to the Austroalpine Superunit and consists of crystalline rocks that have undergone several metamorphic events since their formation during the Precambrian^12^. Lithologies in the valleys include amphibolites, different types of metasediments, and gneisses^13^. Glaciers in the region are temperate and are sensitive to climate oscillations (Fig. 2). Over the past 150 years, rapidly increasing greenhouse gas emissions and resulting warming have led to the melting of glacial ice across the planet. In the Silvretta region, the ice-covered area has decreased to 32 ± 2% of the extent of the Little Ice Age (LIA; ca. 1250–1850 CE) in the reference years 2017/2018 (Fig. 1b)^14^. Jamtal glacier—the main ice body at Jamtal—has retreated to a position ca. 2 km upstream from the LIA maximum. In 2018, it covered approximately 2.8 km^2^ with its terminus at an altitude of ca. 2410 m a.s.l. Glaciers at Fimbatal have largely disappeared today, with only a few small patches of dead ice left in the uppermost sections of the valley (Fig. 1b). In both valleys, rock glaciers are present at altitudes higher than 2400 m a.s.l. In deglaciated sections of the valleys, sequences of lateral and terminal moraines are preserved that evidence stable ice margins of the past and promise insights into periods when climate conditions were favorable for larger glaciers (Figs. 3, 4, 5, Fig. S2).

## Results

**Figure 6:**
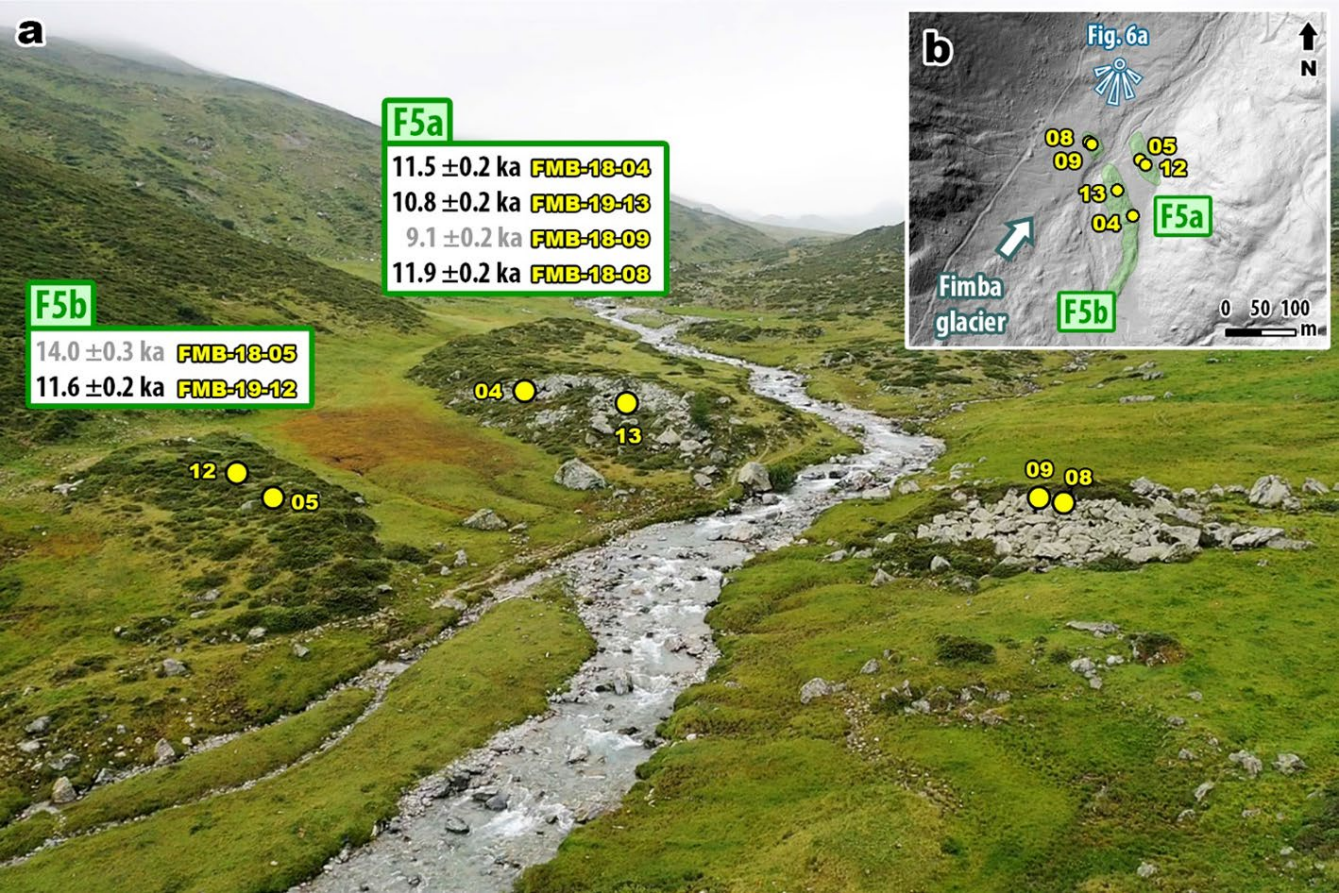
Photograph and map of sampled valley sections at Fimbatal. **(a)** Photograph of F5a and F5b moraines indicating stable termini of Fimba glacier toward the end of the YD at an elevation of ca. 2040 m a.s.l.; outliers are colored in gray. **(b)** Map showing sample and landform locations at Jamtal (DEM provided by Land Tirol—www.tirol.gv.at/als, CC BY 4.0; the map was created using ArcGIS v10.7).

A total of 15 rock samples were collected from moraines at Jamtal (n = 9) and Fimbatal (n = 6). Ages are stratigraphically in order and are presented from old to young, beginning with the Jamtal (JAM) record, followed by the Fimbatal (FMB) record. ^10^Be analytical data and boulder ages are presented in Table 1 and in Figs. 3, 5 and 6 as well as in the supplement (“Sections 1 and 2”).

**Table 1:** The ^10^Be analytical data and corresponding exposure ages of Jamtal and Fimbatal samples.

Sample ID	Latitude [DD]	Longitude [DD]	Elevation [m a.s.l.]	Av. thickness [cm]	Shielding factor	Quartz mass [g]	^9^Be carrier [g]	^10^Be/^9^Be ratio ± 1σ analytical unc.(10^–14^)				^10^Be atoms ± 1σ analytical unc. [atoms]			^10^Be conc. ± 1σ analytical unc. [atoms/g qtz]			^10^Be exposure age ± 1σ analytical unc. and carrier unc. [years]		
**F5b**																				
FMB-18–05	46.9436	10.2698	2040	2.1	0.9677	4.5939	0.1821	10.26 ± 0.20 (2.0%)				1,283,934 ± 25,261			278,524 ± 5480			14,000 ± 280		
FMB-19–12	46.9435	10.2699	2041	1.7	0.9702	10.6165	0.1789	19.79 ± 0.37 (1.9%)				2,440,545 ± 45,594			229,294 ± 4284			11,590 ± 220		
**F5a**																				
FMB-18–04	46.9429	10.2697	2044	1.8	0.9709	42.9287	0.1789	79.07 ± 1.68 (2.1%)				9,716,105 ± 205,863			226,122 ± 4791			11,460 ± 240		
FMB-18–08	46.9438	10.2688	2043	2.0	0.9675	25.5955	0.1787	48.96 ± 0.91 (1.9%)				6,008,892 ± 111,455			234,413 ± 4348			11,920 ± 220		
FMB-18–09	46.9438	10.2689	2042	2.1	0.9703	23.8501	0.1790	34.29 ± 0.64 (1.9%)				4,215,324 ± 79,209			176,366 ± 3314			9100 ± 170		
FMB-19–13	46.9432	10.2694	2044	1.6	0.9728	10.5569	0.1758	18.60 ± 0.35 (1.9%)				2,254,712 ± 42,127			212,986 ± 3979			10,760 ± 200		
**J7**																				
JAM-18–11	46.8904	10.1871	2520	1.3	0.9634	9.2555	0.1806	30.46 ± 0.56 (1.9%)				3,778,652 ± 70,081			407,782 ± 7563			14,710 ± 270		
JAM-19–19	46.8904	10.1875	2522	2.0	0.9607	10.6142	0.1798	34.00 ± 0.64 (1.9%)				4,214,518 ± 78,808			396,476 ± 7414			14,420 ± 270		
**JAM**-20–23	46.8904	10.1872	2521	2.9	0.9620	11.3598	0.1807	35.08 ± 0.71 (2.0%)				4,383,259 ± 88,749			385,607 ± 7807			14,130 ± 290		
**J6**																				
JAM-18–13	46.8901	10.1838	2432	3.4	0.9781	16.5606	0.1806	44.59 ± 0.83 (1.9%)				5,532,868 ± 103,026			333,831 ± 6216			12,880 ± 240		
JAM-19–20	46.8893	10.1865	2445	1.5	0.9604	10.6338	0.1801	26.96 ± 0.50 (1.9%)				3,347,499 ± 62,609			314,211 ± 5877			12,120 ± 230		
**J5**																				
JAM-20–24	46.8895	10.1829	2380	2.4	0.9779	6.8428	0.1805	15.93 ± 0.30 (1.9%)				1,987,961 ± 37,023			290,103 ± 5403			11,620 ± 220		
JAM-20–25	46.8893	10.1835	2389	1.7	0.9768	10.9885	0.1798	26.46 ± 0.52 (2.0%)				3,289,758 ± 64,839			299,123 ± 5896			11,830 ± 230		
**J3-4**																				
JAM-20–26	46.8898	10.1740	2065	1.8	0.9412	11.5032	0.1796	18.81 ± 0.40 (2.1%)				2,335,435 ± 49,824			202,777 ± 4326			10,490 ± 220		
JAM-20–27	46.8908	10.1733	2047	1.4	0.9499	11.5197	0.1803	20.97 ± 0.39 (1.9%)				2,613,912 ± 48,590			226,661 ± 4213			11,650 ± 220		

Samples were analyzed at the CAMS–LLNL. All samples were measured against the 07KNSTD3110 standard with a ratio of 2.85 × 10^–12^^25^. One to two procedural blanks were processed with each batch of samples with ratios ranging from 2.3 to 8.4 × 10^–16^ (Table S2). The ^10^Be background contamination measured in the blanks was subtracted from the samples. Exposure ages were calculated with the calculator formerly known as CRONUS–Earth online calculator v3^26^, using the Swiss ^10^Be production rate^27﻿^ and the ‘Lm’ scaling scheme. Ages are calculated relative to the sampling year denoted by the first number in the sample ID and are rounded to the nearest 10 years. Uncertainties of boulder ages include the 1σ analytical error and a 1% uncertainty on the carrier concentration.

At Jamtal, our focus was on a steep (> 30°) valley flank that features a right-lateral moraine set that was shaped by a former tributary glacier (Futschöl glacier; Figs. 3a,b, Fig. S2, and S3). The uppermost landform selected for ^10^Be surface exposure dating is J7 at an elevation of ca. 2520 m a.s.l. (Fig. 4a). The three sampled boulders rest on a till-covered lineament and yield a landform age of 14.4 ± 1.0 ka (Fig. S1a). Around 80 m below J7, at ca. 2440 m a.s.l., a sharp-crested moraine (J6) was deposited that consists mainly of fine sediments (Fig. 4b) and features two boulders that qualified for sampling. Corresponding ages are 12.9 ± 0.2 ka and 12.1 ± 0.2 ka. The ice margin geomorphology another level below denoted as J5 (Fig. 4d), differs from J6 in the absence of a distinct ridge and in the abundance of boulders, of which two were selected for dating and yield ages of 11.6 ± 0.2 ka and 11.8 ± 0.2 ka. Two boulders in the main valley embedded in lateral and in terminal moraines (J3–4, Fig. 3c,d) give ages of 10.5 ± 0.2 ka and 11.7 ± 0.2 ka and extend the Holocene moraine chronology published for the valley by Braumann, et al.^9^ (Fig. 5).

At Fimbatal, samples were collected from two adjacent latero-frontal moraine ridges (F5a and F5b) deposited at ca. 2040 m a.s.l. (Fig. 6). The outer ridge (F5b) consists of primarily fine sediments and is overgrown by vegetation. It is preserved only on the east side of the creek and has few boulders exposed, two of which were sampled and yield ages of 14.0 ± 0.3 ka (FMB-18-05) and 11.6 ± 0.2 ka (FMB-19-12). Although FMB-18-05 agrees within errors with boulder ages along J7 at Jamtal, we reject the age as an outlier due to its stratigraphic position. A terminal moraine at Fimbatal that is equivalent to the lateral ice-margin position at Jamtal should be positioned further downstream. FMB-18-05 probably overestimates the age of F5b, most likely due to inheritance from one or multiple earlier exposure events.

The inner ridge (F5a) is preserved on both sides of the creek and exhibits a blocky structure in its frontal section caused by the outwash of fine-grained material. Exposure ages of the four boulders along F5a range between 11.9 ± 0.2 ka and 9.1 ± 0.2 ka. The age of FMB-18-09 (9.1 ± 0.2 ka) falls within a period during the Holocene when most glaciers had probably retreated inboard the subsequent LIA ice margin^19,28,29^. For reference, the LIA terminal moraine in this valley is located at an elevation of ca. 2520 m a.sl., i.e,. around 500 m higher and almost 7 km upstream of F5a/b (Fig. 1b). These vertical and lateral distances cannot be reconciled with the age and location of FMB-18-09. Therefore, we discard the age for our interpretation. Ages of the remaining moraine boulders featured by F5a in concert with the age of FMB-19-12 of F5b correlate with the moraine age of J5 at Jamtal. The Fimbatal geochronology makes a case for two closely spaced stable ice margins toward the end of the YD.

## Discussion

Boulder ages in both valleys fall into three periods: (1) the Oldest Dryas to Bølling (J7), (2) the Younger Dryas (J6, J5, F5a, and F5b), and (3) the Early Holocene (J3–4), and are discussed in this order.

### Pre-Bølling to Bølling transition

The deposition of boulders featured by landform J7 (14.4 ± 1.0 ka) occurred during a period when regional climate was transitioning from stadial to interstadial conditions, i.e., from the Oldest Dryas cold phase to the Bølling warm phase. We review the deglaciation of the European Alps in the millennia before the exposure of J7 boulders to place the new moraine records at Jamtal and Fimbatal in a coherent temporal and spatial context of the Alpine LG.

**Figure 7:**
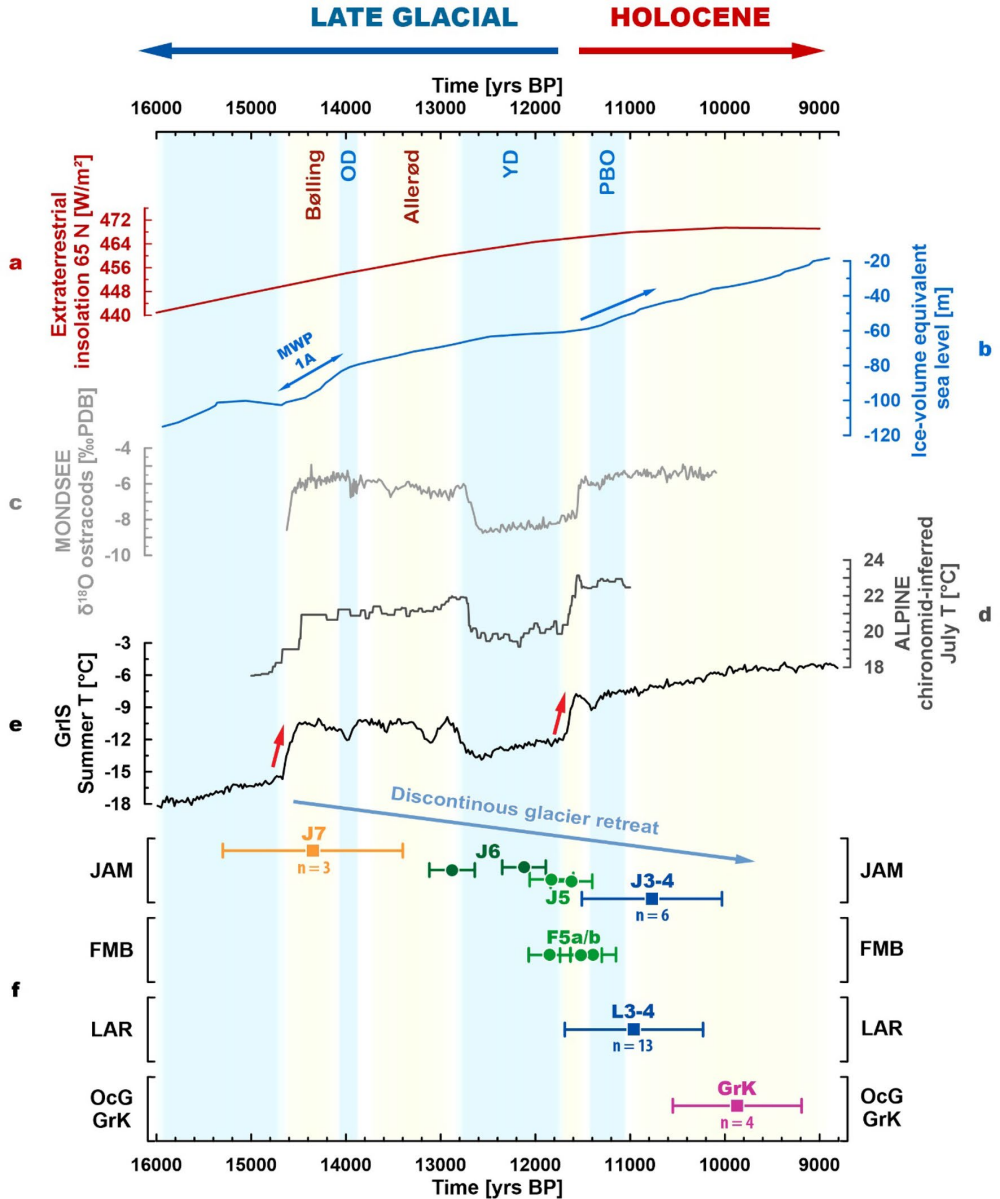
Silvretta mountain glacier records correlated with solar insolation, sea-level rise, and climate proxy records from different regions in the Northern Hemisphere. **(a)** Insolation at 65 N^40^ gradually increases during the LG and is a natural forcing for climate warming during this period. **(b)** Global sea-level rise due to deglaciation of the cryosphere^41^; note increased rate of change between 14.5 and 14 ka, and 11.4 ka onwards. **(c)** Local high-resolution ostracod record from lake Mondsee^42^. **(d)** Chironomid-inferred summer temperature stack record for the Alpine region^43^. **(e)** Summer temperatures from the Greenland Ice Sheet (GrIS) reconstructed based on oxygen isotopes in Greenland ice cores^44^. Temperatures reconstructed using different methods and representing different spatial scales in the Northern Hemisphere agree well and capture the YD stadial as well as the two bracketing episodes of rapid climate warming, the pre-Bølling to Bølling transition and the YD–EH transition. **(f)** Glacier stabilization during general LG warming is indicated by moraines deposited during Oldest Dryas to Bølling transition (J7), the YD (Egesen moraines J6, J5, and F5a/b), and the EH (J3–4 and L3–4) in this study and Braumann, et al.^9^. By 10 ka, Silvretta glaciers have retreated to positions that resemble (subsequent) LIA extents indicated by the ^10^Be moraine record in the adjacent Ochsental^19^. Moraine ages (n ≥ 3) are shown with rectangles and individual boulder ages with circles. Kernel plots of moraine ages are provided in the supplement (Fig. S1). For the spatial context of the displayed ^10^Be records, see Figs. 1b and 8.

During the Gschnitz stadial, a well-documented post-LGM glacier readvance around 17–16 ka, the uppermost sections of the main alpine valleys and their tributary valleys were glaciated^30^. This phase of readvance that is often associated with Heinrich event 1 in the North Atlantic^31^ was followed by a period of still relatively low mean annual temperatures but slightly increasing summer temperatures paralleling increasing solar insolation (Fig. 7a,e). Despite the probably cold winters, glaciers in the Alps responded to the warmer summers after 16 ka^32^ and retreated to higher elevations. Several recent studies have investigated the pace of post-LGM deglaciation in selected inner-alpine pass regions by applying surface exposure dating to bedrock sections along transects^33–39^. Results indicate that major ice transfluence zones, for instance, the Gotthard, the Grimsel, and the Simplon pass became ice-free between ca. 16 and 14 ka. However, local glaciers with extents that exceeded the subsequent YD glaciation may have been present until the Bølling. At Jamtal, the J7 landform indicates a lateral ice margin position outboard the Egesen moraines around 14.4 ± 1.0 ka, which confirms the timing of ice decay reconstructed in these deglaciation studies.

Depending on the geomorphological interpretation of the J7 landform, different ice dynamics and thus different paleoclimatological scenarios are conceivable to explain the deposition of the sampled boulders. The rounded edges of J7 boulders, together with their position at a lateral distance of approximately 40–50 m from the valley flank, indicate glacial processes that led to their deposition (Figs. S4, S6 and S8). Their alignment along the crest of J7 suggests their accumulation along a stable ice margin, hence during glacier stagnation. In this case, the landform age points towards a short-lived cold event around 14.4 ± 1.0 ka. The location of the landform at a—for a lateral moraine—high elevation (relative to the presumable equilibrium line altitude)^45,46^, however, challenges this interpretation. The damming of the tributary (Futschöl) glacier by the main (Jamtal) glacier (Fig. 3b and Fig. S2) in combination with ideal preservation conditions on top of the flat bedrock section where J7 was found, may explain the presence of a lateral moraine at an unusually high altitude. An alternative interpretation is that the boulders have melted out during glacier retreat. Then, J7 would not mark a stable ice margin and imply cooling but instead suggest continuous warming. In our view, both geomorphological interpretations are plausible and document glacier extents during the Oldest Dryas to Bølling transition that definitely exceeded YD limits.

Since the hypothesis of glacier stagnation after the Gschnitz and before the YD stadial has been proposed and discussed in the past in the context of the European Alps^7,47–49^, we examine the possibility of J7 being a moraine, including the paleoclimatological implications in more detail. LG moraines provide geomorphological evidence of discontinuous deglaciation. Therefore, their identification in alpine valleys allows inferences about cold phases that interrupted the general post-LGM warming trend. The relative moraine stratigraphy of the Alpine region is based on this approach and suggests up to six more or less recognized stadials^47,48^, most prominently the above described ‘Gschnitz’ stadial and the subsequent upstream Egesen stadial. Egesen moraines are distinct multi-ridge structures ubiquitous in high-alpine valleys and are accepted as the morphostratigraphical equivalent of YD cooling^7,50,51^. A less conspicuous stadial, proposed as a stable ice margin between the Gschnitz and the Egesen moraines, is the putative ‘Daun’ stadial. Daun moraines are described as recessional moraines, with less pronounced crests, often with few boulders, and sometimes affected by solifluction. They are assumed to indicate pre-Bølling glaciers at local, inner-alpine locations, closer to the subsequent Egesen moraines than to Gschnitz moraines. The presence of corresponding moraines in the Alps is sparse, hence its acknowledgment as an independent stadial that is discernable at several sites across the Alps remains controversial^49^. The underrepresentation of presumable Daun moraines in geochronological studies in the Alps may be owed to the absence of a corresponding cold snap causing moraine formation. The above-described scenario of J7 boulders having melted out during Bølling warming-driven glacier retreat supports this hypothesis. The—compared to Egesen moraines—unspectacular morphology of putative Daun moraines in tandem with poorer preservation and fewer datable boulders may also be an explanation for the scarcity of moraines identified and dated in between the Gschnitz and the Egesen moraines in the Alps so far. Other factors that influence the formation and preservation of moraines are microclimate, debris cover in the ablation zone, and erosional processes in the catchment. However, any attempt to correlate J7 with the traditional Daun stadial first requires the geomorphological evaluation and dating of the type locality in the Austrian Stubai Alps^52^. Therefore, in the following sections, we refer to J7 and equivalent landforms identified in other alpine valleys as (pre-)Bølling moraines.

In a geochronological study at the Great Aletsch glacier, Schindelwig, et al.^10^ investigated an ice margin indicative of glacier extents that exceed the Egesen extent. They obtained a (recalculated) age of 14.4 ± 0.7 ka (their sample VBA-7) for a boulder sitting on top of a bedrock section that deglaciated at the same time. The authors interpret the site as an LG ice margin of the Great Aletsch glacier. Böhlert, et al.^38^ yielded a (recalculated) age of 15.2 ± 1.9 ka for a boulder (their sample VM7) embedded in a lateral moraine outboard the presumable Egesen moraine at Val Mulix (Switzerland) and ascribed the landform to the Daun stadial. Rolland, et al.^39^ combined the analysis of proglacial lake sediments in the Argentera-Mercantour Massif with exposure dating of glacial features in the region and identified a LG moraine that was deposited ca. 14.6 ± 0.9 ka (Vens moraine, n = 3). These dated boulders and landforms, albeit limited in number, are in good agreement with the age of J7 and suggest moraine formation between ca. 16 and 14 ka in the Alps.

Considering the uncertainty calculated for the age of J7 (14.4 ± 1.0), the landform could also have been deposited at the onset of the Bølling interstadial, during the Older Dryas cold snap (ca. 14 ka), or during the subsequent Allerød interstadial (until 12.9 ka). However, we emphasize that the most likely age of deposition is 14.4 ka, hence the earliest part of this warm phase (Fig. S1a). The B–A interstadial was identified in numerous climate archives in the Northern Hemisphere^42,43,53–55^. Its onset around 14.6 ka is characterized by an abrupt temperature increase that induced substantial changes in environmental and vegetational conditions^56,57^. Summer temperatures increased by several degrees in the Alps^43,54^, transitioning from cooler stadial to warmer interstadial levels (Fig. 7c,d). Glaciers responded to this warming and might have retreated to the highest cirques of the Alps or disappeared entirely during the B–A temperature plateau. The demise of glaciers during this interstadial appears in conflict with concurrent moraine formation of the J7 landform outboard the subsequent Egesen ice margin. Yet, weakening of the Atlantic Meridional Overturning Circulation (AMOC) may explain centennial-scale cooling during general warming and contemporaneous stabilization or readvance of glaciers in the European Alps. Freshwater input into the North Atlantic Ocean during the deglaciation of adjacent ice sheets has the potential to decelerate or even shut down the warm northwards flowing AMOC limb and temporarily reduce heat transport to Northern Europe^58,59^. Evidence of repeated freshening of ocean water between 15.8 and 12.6 ka has been detected in a sediment core south of Iceland and has been linked to the deglaciation of the Laurentide ice sheet^60^. A reduction of poleward heat transport due to AMOC weakening may have led to centennial-scale episodes of cooling detected in Northern and Central Europe, such as the Older Dryas and the later Gerzensee oscillations (13.3–13.0 ka)^56^, and perhaps also to a cold spell, a few centuries earlier suggested by a potential J7 moraine.

The link between the deglaciation of the Laurentide ice sheet, resulting in freshwater input into the North Atlantic, and cooling in Europe has been suggested as an explanation for abrupt centennial-scale cooling in the context of the YD-Holocene transition^61,62^. Moraine formation as a result of this teleconnection on the LG to Holocene time scale has recently been proposed by Young, et al.^63^ for the Greenland ice sheet, by Protin, et al.^8^ for mountain glaciers in the French Alps, by Braumann, et al.^9^ for the Austrian Alps, and by Jomelli, et al.^64^ for glaciers in the tropical Andes and the greater North Atlantic region. Comparing the warming during the pre-Bølling to Bølling transition and during the YD–Holocene transition (Fig. 7e), we tentatively suggest brief episodes of AMOC slowdown and subsequent cooling in Europe for both periods. Even though the causation between Bølling warming, freshwater input into the Atlantic, and the AMOC circulation is elusive, we note that the timing of Bølling warming parallels MWP1A, a sea-level rise of about 12–22 m within a few centuries (Fig. 7b)^41^. The source of MWP1A remains under debate and has often been attributed to the Antarctic ice sheet alone^65^. However, recent sea-level fingerprinting and ice sheet modeling studies suggest that the melting of ice sheets in the Northern Hemisphere likely has contributed to massive sea-level rise at that time and may have caused centennial-scale cold lapses^66–68^.

Both scenarios—moraine deposition prior to or during the Bølling—are plausible, but more direct age data covering that period are needed to better constrain moraine deposition and thus cooling between 16 and 14 ka in the European Alps. Interestingly, when comparing the (pre-)Bølling moraine age of J7 with mountain glacier records beyond the Alps, we find similar intervals of moraine formation along (former) ice sheets and in alpine catchments in Norway^69,70^, in Patagonia^71–75^ and New Zealand^76,77^, and the Himalaya region^78^. In the Southern Hemisphere, the climatic explanation for this phase of glacier advance is the Antarctic Cold Reversal (ACR; 14.5–12.7 ka), a millennial-scale cold phase documented in Antarctic ice cores^79^. The extent to which this cold phase propagated from Antarctica and the Southern Hemisphere further to the North remains controversial, but we note that moraine formation indicated by J7 falls within the early phase of the ACR.

### Younger Dryas: Egesen moraines

The next LG time slice that is captured by the new moraine chronologies of both valleys is the YD period. We interpret moraines J6 and J5 at Jamtal and F5a and F5b at Fimbatal as Egesen moraines that portray the fine structure of glacier advance or stabilization during this final stadial before Holocene warming (Fig. 7e). The J6 moraine at Jamtal indicates a stable ice margin in the YD and likely marks the maximum glacier extents during the first half of this cold phase. The lower J5 moraine and F5a/b at Fimbatal delimit glacier extents toward the very end of the YD. Exposure ages from these landforms confirm relative age estimates from a previous study in the Silvretta region focused on geomorphology and stratigraphy^80^.

The Egesen moraine sequence at Jamtal shows that the ice surface lowered by about 55 m from J6 (ca. 2440 m a.s.l.) to J5 (2385 m a.sl.) within a few centuries (Fig. 4c), hence supporting the hypothesis that climate conditions became gradually milder through the YD^81^. Glacier retreat through the YD with intermittent phases of glacier stabilization is observed in the European Alps^10,82,83^ and in other glaciated regions of the Northern Hemisphere^69,84^, and coincides with slightly increasing summer temperatures (Fig. 7e)^42,43,44^. The mountain glacier record of the Southern Hemisphere indicates moraine deposition through glacier retreat during the same time interval^74,85^, which corroborates the gradual expansion of YD cooling towards the Southern Hemisphere^86^.

### Early Holocene

**Figure 8:**
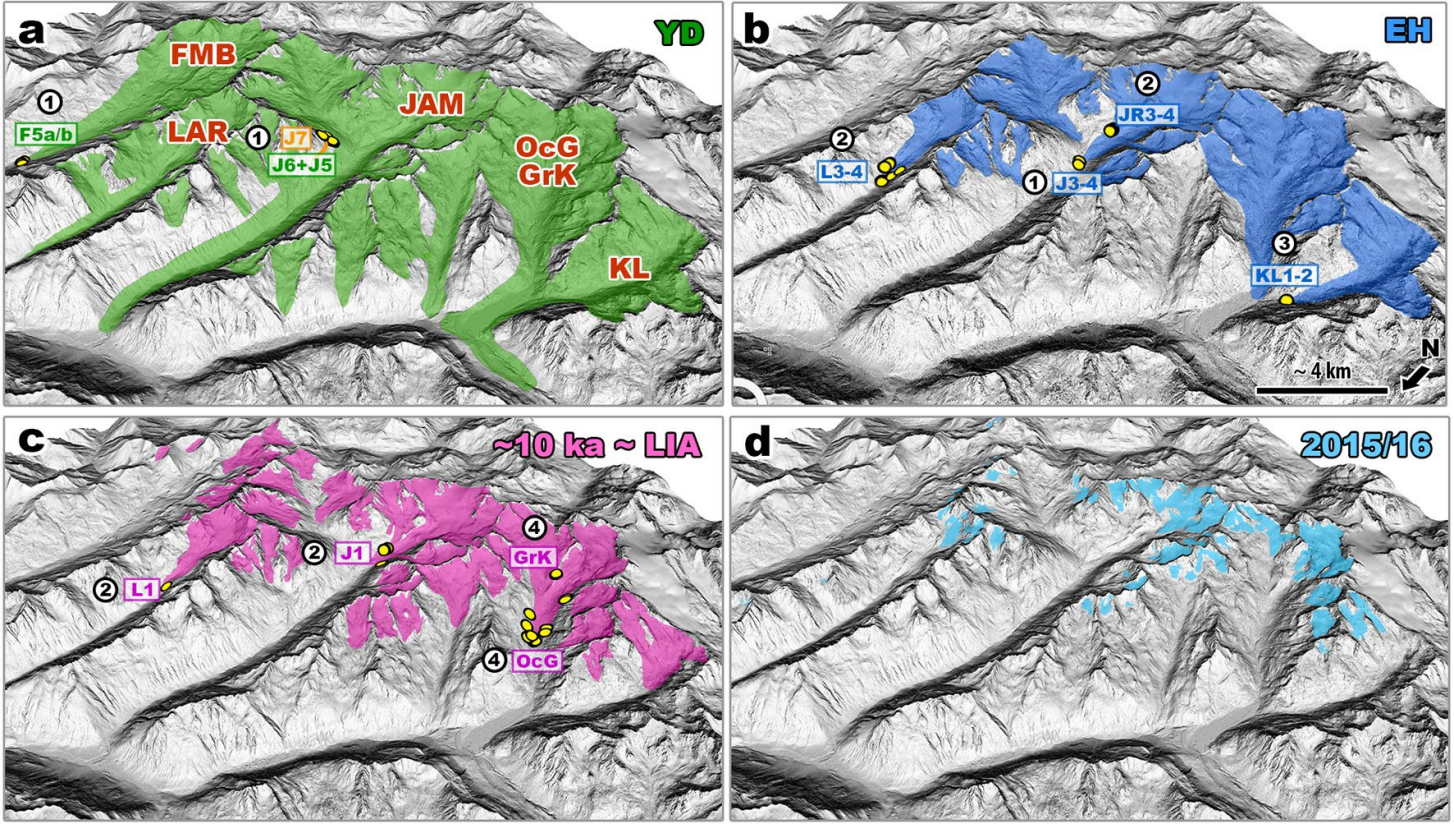
Possible extents of Silvretta glaciers during the **(a)** the later YD, **(b)** the EH, **(c)** around 10 ka and during the LIA, and **(d)** in 2015/16 CE. Positions of dated boulders are indicated with yellow symbols across all valleys and time slices and are published in this study (#1), in Braumann, et al.^9^ (#2), in Moran, et al.^18^ (#3), and in Braumann, et al.^19^ (#4). IDs of corresponding moraines are adopted from original publications and are shown in rectangles. Ice margins positions with geochronological data available (yellow circles) are reliable. All other ice margins in (**a**) and (**b**) are estimates and are based on Hertl^80^. LIA and modern ice margins are taken from the Austrian and the Swiss glacier inventories, respectively^15,16^. The Jamtal moraine record in conjunction with previously published moraine records of the region indicates that the transition from YD to EH ice extents occurred within a few centuries. DEMs provided by Land Tirol (www.tirol.gv.at/als; CC BY 4.0) and Land Vorarlberg (http://vogis.cnv.at/atlas/init.aspx; CC BY 4.0). The map was created using ArcGIS Pro.

Ice-surface lowering during the YD from J6 to J5 was rapid, but the rate of glacier change during the transition from the YD to the EH was even faster. The downwasting of Jamtal glacier and its tributary Futschöl glacier during this period is best illustrated by comparing moraine segments J5 and J3–4 (Fig. 5). Even though the boulder ages of J5 and JAM-20-27 (J3–4) are statistically indistinguishable, the associated landforms indicate very different glacier positions (Fig. 8a,b). J5 marks the right-lateral ice margin of the tributary (Futschöl) glacier when it still converged with the main (Jamtal) glacier. In turn, J3–4 indicates much smaller glacier extents when the main and tributary glaciers were separated, implying deglaciation of the valley flank within a few centuries (Fig. 3a and Fig. S2). The new early Holocene terminus of Jamtal glacier marked by JAM-20-27 (Fig. 3d) is located only around 900 m outboard the Holocene/LIA moraine. J3–4 is interpreted as the equivalent of the right lateral early Holocene moraine set (JR3–4) that was mapped and dated in a previous study^9^ (Fig. 5), which in concert with the adjacent EH Laraintal chronology, indicates moraine deposition and thus glacier stabilization ca. 11.0 ± 0.7 ka (Figs. 7f, 8b, and Fig. S1). The timing of moraine formation overlaps with the Preboreal Oscillation, a centennial-scale cold pulse in (Northern) Europe, which was likely caused by AMOC weakening due to freshwater input into the Atlantic—the same mechanism that was tentatively proposed earlier for the deposition of J7 during the early Bøllingn. Similar to the (pre-)Bølling and YD period, the synchronicity of mountain glacier stabilization during the EH is observed in glaciated regions of both hemispheres^63,87–90^.

Silvretta glaciers probably remained outboard their subsequent LIA ice margins for the next several centuries but retreated to LIA-like configurations around 10 ka, which is shown based on a ^10^Be moraine chronology from the adjacent Ochsental (Fig. 8c)^19^. Throughout the rest of the Holocene, they probably oscillated inboard the 10 ka limits (e.g., Fig. 8d) with advances(s) to the 10 ka-position possibly during the Neoglacial and certainly during the LIA^15^.

### Broader relevance of the new moraine chronologies

The ^10^Be datasets from the Silvretta Massif are, to date, the most detailed cosmogenic-nuclide-based mountain glacier records in the Eastern European Alps. They pinpoint the timing of moraine formation in the region around 14.4 ± 1.0 ka, during the YD between 12.9 ka and 11.7 ka, and during the Early Holocene around 11.0 ± 0.7 ka^9^ and around 9.9 ± 0.7 ka^19^. Thus, the new glacier reconstructions unravel short-lived climate oscillations during rapid warming phases and point towards feedbacks related to AMOC variability. The Silvretta mountain glacier records contribute to our understanding of the climate system transitioning from glacial to interglacial conditions, provide valuable constraints for modeling (paleo-)glaciers and address the resilience of mountain glaciers under rapidly warming climate conditions. The response of Silvretta glaciers to natural warming during recent geological periods shows their sensitive response to climate change and places the magnitude and impact of anthropogenic climate forcing in a natural context.

A comparison of moraine records of ice sheets and alpine glaciers on a global scale suggests that glaciers in both hemispheres deposited moraines around 15–14 ka, during the YD, and during the EH. The next step is to investigate whether these similarities are coincidental or due to large-scale climatic forcing.

## Methods

### Geomorphological mapping

Hertl^80^ developed a relative moraine stratigraphy for the region this work is based on. The pre-existing geomorphological maps were updated and supplemented with information gained during several field campaigns in the summers of 2018 to 2021, from remote sensing data^91,92^ and drone imagery. Glacier reconstructions of Jamtal glacier for the Holocene, including corresponding maps, are presented in Braumann, et al.^9^. This study extends the Jamtal glacier chronology into the LG and focuses on landforms that evidence glacier oscillations during this period by mapping and dating ice margins outboard the Holocene moraines. Reconstructions of paleoglaciers are complemented with glaciological data of modern, annual- to decadal-scale glacier change, including observations of glacier mass balances, front variation, and ice-covered area^14,20,93,94^.

### Surface exposure dating with ^10^Be

When fresh quartz-containing rock surfaces are exposed to cosmic radiation, the production of the cosmogenic radionuclide ^10^Be begins according to the nuclide-specific production rate (ca. 4 atoms g^−1^ year^−1^ at sea level and high latitude). The longer a surface has been exposed, the more ^10^Be has accumulated^95^. Thus, the nuclide content in rock surfaces is a function of exposure time. This principle is used in the application of surface exposure dating to glacial landforms. A moraine boulder sampled for ^10^Be analysis has ideally been eroded from bedrock beneath the glacier, has been transported sub- or englacially, and has finally been deposited at a stable ice margin. It has not been exposed to cosmic radiation prior to its deposition along the ice margin, so its radionuclide inventory is ‘zeroed’. If these assumptions are valid, the ^10^Be inventory measured in a rock surface after exposure will produce an age that reflects the boulder’s melt out of glacial ice, or in other words, the onset of ice retreat, hence warming.

Rock samples were collected using an electric saw, and hammer and chisel. The sample location was measured using a hand-held GPS device. Strike and dip angles of sampled rock surfaces were quantified using a geological compass. Samples were preferably collected from boulder tops at windswept locations to minimize shielding effects due to snow and/or sediment cover. We avoided surfaces affected by exfoliation and prioritized boulder locations with striations preserved on the surface, which indicates low postglacial erosion. For more details on our sample selection criteria, we refer to Braumann, et al.’s supplement, their Table S1.

Mechanical sample preparation was accomplished at the Department of Lithospheric Research of the University of Vienna and at the Lamont-Doherty Earth Observatory (LDEO). Whole-rock samples were crushed to grain sizes between 63 and 500 µg using a mill or a jaw crusher. Mineral separation strategies included magnetic separation, boiling with phosphoric acid, froth flotation, density separation and repeated (> 3) leaches with hydrofluoric acid and nitric acid at concentrations between 1 and 5 %^96^. Purified quartz yields, the target mineral for ^10^Be extraction, ranged from 1.1 % to 11.3 % (Table S1). Samples were processed in four batches (Table S2) with quartz weights ranging from 4.5939 to 42.9287 g (Table 1). All samples were spiked with the LDEO ^9^Be carrier (#7) made of deep-mine beryl, which has a concentration of approximately 1000 ppm. The extraction of ^10^Be was accomplished following the LDEO protocol^97^. Isotope ratios in samples (^10^Be/^9^Be) were measured at the Center for Accelerator Mass Spectrometry (CAMS) facility, Lawrence Livermore National Laboratory (LLNL) using the 07KNDSTD3110 standard with a ^10^Be/^9^Be ratio of 2.85 × 10^–12^^25^.

Exposure age calculations including statistical outlier identification (Χ^2^ test) were performed using the online calculator formerly known as the CRONUS–Earth online calculator v3^26^. We used the local ‘Swiss’ production rate^27^ and chose the time-dependent ‘Lm’ scaling scheme^98^. In the data presentation and discussion, we distinguish between exposure ages of individual boulders and moraine ages. Boulder ages are reported with 1σ analytical uncertainties and a 1 % error on the carrier concentration but without uncertainties on the production rate as this source of uncertainty is constant for all boulders sampled from the same area. For moraine ages (n ≥ 3), the production rate error is propagated in quadrature to analytical and carrier uncertainties to allow the correlation of landform ages with moraine records from other regions.

## Supplementary Information

Supplementary Information.

## Data Availability

All analytical information associated with cosmogenic nuclide measurements is listed in the tables in the Supplement and will be made available via the ICE-D Alpine database (http://alpine.ice-d.org/).
