# Supplementary material for: Moraines in the Austrian Alps record repeated phases of glacier stabilization through the Late Glacial and the Early Holocene
Source: Sci Rep. 2022 Jun 13;12:9438. doi: 10.1038/s41598-022-12477-x (PMC9192639; doi:10.1038/s41598-022-12477-x)
Supplement: Supplementary file 1 — Supplementary Information. [file 41598_2022_12477_MOESM1_ESM.pdf]

SUPPLEMENT

Moraines in the Austrian Alps record repeated phases of glacier stabilization through the Late Glacial and the Early Holocene

AUTHORS
Sandra M. Braumann[1,2,*], Joerg M. Schaefer[2], Stephanie Neuhuber[1], Markus Fiebig[1]

AFFILIATIONS
[1] *University of Natural Resources and Life Sciences (BOKU), Peter Jordan-Straße 82, A-1190 Vienna, Austria*

[2] *Lamont-Doherty Earth Observatory of Columbia University, Division of Geochemistry, Palisades, NY 10964, USA*

* CORRESPONDING AUTHOR
Address: Sandra M. Braumann
BOKU University
Peter-Jordan Straße 82
1190 Vienna
Austria

E-Mail: sandra.braumann@boku.ac.at

Phone: +43 1 47654 87207

CONTENT

1 | Quartz content in rock samples **p2**

2 | Organization of ^{10}Be sample batches including procedural blanks **p3**

3 | Kernel plot of J7 **p4**

4 | Geomorphological observations and sample documentation **p6**

Jamtal samples (JAM) p6

Fimbatal samples (FMB) p15

References **p21**

1 QUARTZ CONTENT IN ROCK SAMPLES

Table S 1: Quartz yields of rock samples collected from Jamtal (JAM) and Fimbatal (FMB); values range between 1.1 and 11.3% with a median of 5.2%.

Sample ID	Start weight [g]	Quartz yield [g]	Quartz yield [%]
**JAM-18-11**	481	9.01	1.9%
**JAM-18-13**	685	16.74	2.4%
**JAM-19-19**	412	40.13	9.7%
**JAM-19-20**	363	18.97	5.2%
**JAM -20-23**	436	26.90	6.2%
**JAM-20-24**	465	7.02	1.5%
**JAM-20-25**	440	11.20	2.5%
**JAM-20-26**	317	35.70	11.3%
**JAM-20-27**	462	25.05	5.4%
**FMB-18-04**	419	42.93	10.2%
**FMB-18-05**	409	4.59	1.1%
**FMB-18-08**	420	25.79	6.1%
**FMB-18-09**	356	23.85	6.7%
**FMB-19-12**	476	19.16	4.0%
**FMB-19-13**	647	16.41	2.5%
		**Maximum**	**11.3%**
		**Minimum**	**1.1%**
		**Median**	**5.2%**

2 ORGANIZATION OF ^{10}BE SAMPLE BATCHES INCLUDING PROCEDURAL BLANKS

Table S 2: A total of 15 rock samples was processed according to the LDEO protocol [1,2]. Samples were processed in four batches, each of them including one to two procedural blanks (in blue font). The numbers of atoms counted in blanks that were processed with each batch were subtracted from the total number of atoms in each sample of the same batch. If two blanks were available (batches 3 and 4), the average of both blanks was used for blank correction. Blank correction ranged from 0.1% to 0.3% depending on the total number of ^{10}Be atoms in the samples. Concentrations of the LDEO ^{9}Be carriers are corrected for evaporation.

Batch #	Sample ID	^{10}Be/^{9}Be AMS ratio	1σ anal. unc. ^{10}Be/^{9}Be AMS ratio	1σ anal. unc. ^{10}Be/^{9}Be AMS ratio [%]	Counted ^{10}Be atoms [atoms/sample]	1σ anal. unc. ^{10}Be/^{9}Be [atoms/sample]	Blank correction [%]	Carrier & Concentration [ppm}
3	**Blk1_2019May23**	**8.36E-16**	**2.09E-16**	**25.0%**	**10266**	**2566**	-	**Carrier 7 1028**
	**Blk2_2019May23**	**6.18E-16**	**1.72E-16**	**27.8%**	**7694**	**2135**	-	
	FMB-18-04	7.91E-13	1.68E-14	2.1%	9716105	205863	0.1%	
	FMB-18-08	4.90E-13	9.08E-15	1.9%	6008892	111455	0.1%	
	FMB-18-09	3.43E-13	6.44E-15	1.9%	4215324	79209	0.2%	
4	**BLK1-2019June6**	**4.32E-16**	**1.44E-16**	**33.4%**	**5355**	**1786**	-	**Carrier 7 1028**
	**BLK2-2019June6**	**2.81E-16**	**9.95E-17**	**35.4%**	**3492**	**1236**	-	
	JAM-18-11	3.05E-13	5.65E-15	1.9%	3778652	70081	0.1%	
	JAM-18-13	4.46E-13	8.30E-15	1.9%	5532868	103026	0.1%	
	FMB-18-05	1.03E-13	2.02E-15	2.0%	1283934	25261	0.3%	
6	**BLK3-2020Jan31**	**5.05E-16**	**1.46E-16**	**28.9%**	**6243**	**1804**	-	**Carrier 7 1032**
	JAM-19-19	3.40E-13	6.36E-15	1.9%	4214518	78808	0.1%	
	JAM-19-20	2.70E-13	5.04E-15	1.9%	3347499	62609	0.2%	
	FMB-19-12	1.98E-13	3.70E-15	1.9%	2440545	45594	0.3%	
	FMB-19-13	1.86E-13	3.48E-15	1.9%	2254712	42127	0.3%	
7	**BLK-2021Nov17**	**2.29E-16**	**8.09E-17**	**35.4%**	**2846**	**1008**	-	**Carrier 7 1035**
	JAM-20-23	3.51E-13	7.10E-15	2.0%	4383259	88749	0.1%	
	JAM-20-24	1.59E-13	2.97E-15	1.9%	1987961	37023	0.1%	
	JAM-20-25	2.65E-13	5.22E-15	2.0%	3289758	64839	0.1%	
	JAM-20-26	1.88E-13	4.01E-15	2.1%	2335435	49824	0.1%	
	JAM-20-27	2.10E-13	3.90E-15	1.9%	2613912	48590	0.1%	
	**Blank min.**	**2.29E-16**						
	**Blank max.**	**8.36E-16**						

3 LANDFORM AGE CALCULATIONS

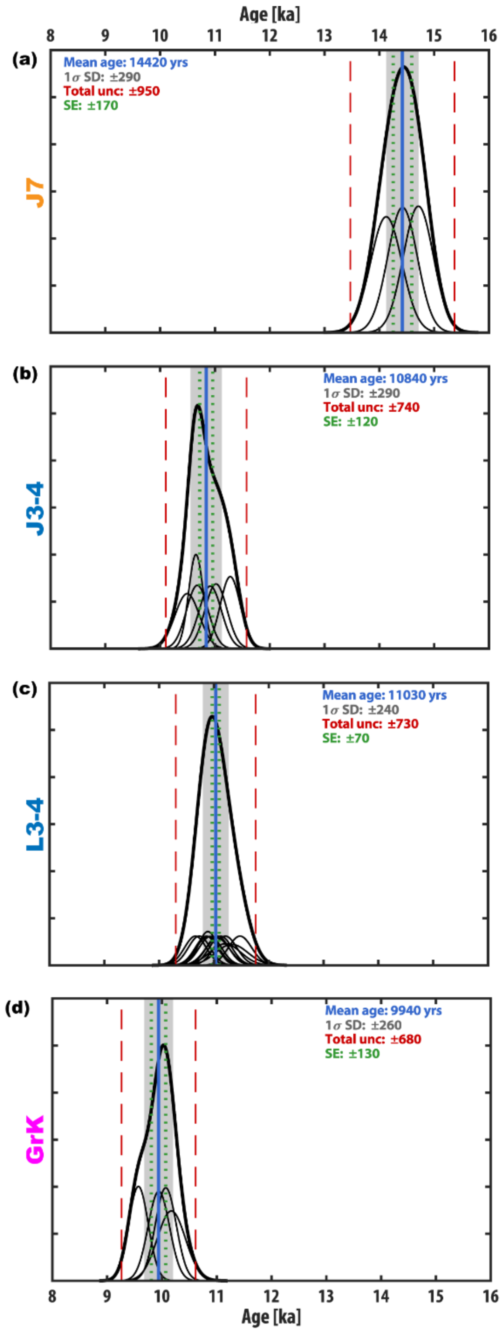

Figure S 1: Kernel plots of moraine ages shown in Figure 6. (a.) J7 (n=3; samples: JAM-18-11, JAM-19-19, JAM-20-23). (b.) J3-4 (n=6; samples: JAM-18-01, JAM-18-02, JAM-18-04, JAM-19-21, JAM-19-22, JAM-20-26). (c.) L3-4 (n=13; samples: LAR-18-10, LAR-19-12, LAR-19-13, LAR-19-14, LAR-19-15, LAR-19-16, LAR-19-17, LAR-19-18, LAR-19-19, LAR-19-20, LAR-19-21, LAR-19-22, LAR-19-24). (d.) GrK (n=4; samples: GrK-17-01, GrK-17-02, GrK-17-03, GrK-17-04). The gray-shaded bars indicate the 1σ standard deviation (SD) of the landform ages calculated from the analytical uncertainties of individual samples. Dashed red lines add the production rate uncertainty and the uncertainty of the carrier concentration to the 1σ SD and show the total uncertainty. Dotted green lines are the standard error (SE), which describes the dispersion of different sample means from the population mean.

4 GEOMORPHOLOGICAL OBSERVATIONS AND SAMPLE DOCUMENTATION

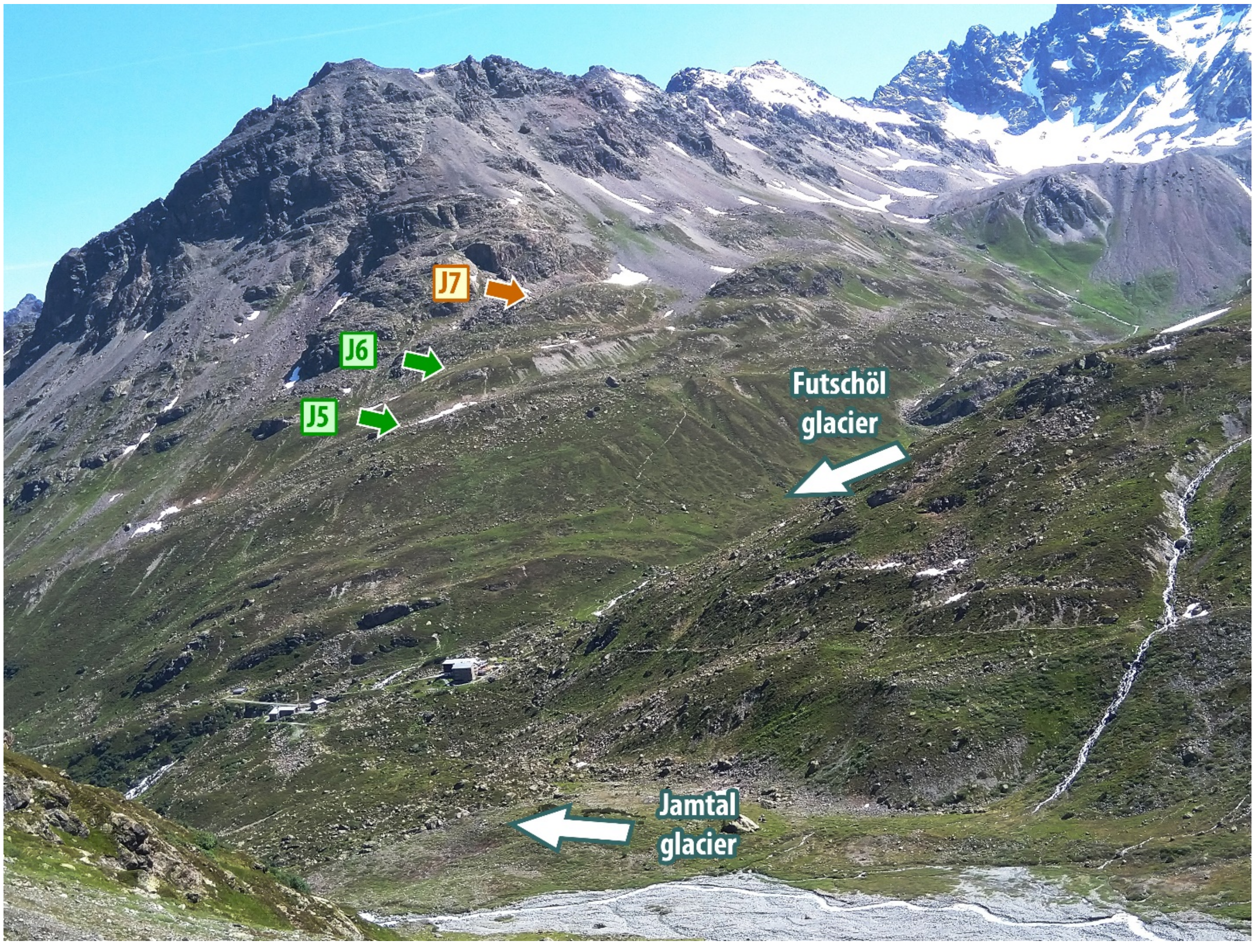

Figure S 2: Confluence zone of the Jamtal glacier and the tributary Futschöl glacier. Lateral moraines J7, J6 and J5 are dated using ^{10}Be surface exposure dating. The resulting ages are stratigraphically in order: Three boulders from J7 produce a landform age of 14.4 ± 1.0 ka falling into the (Pre)-Bølling period. J6 and J5 both date to the Younger Dryas (YD) with the upper ridge indicating an ice margin during the first half of the YD, and the lower ridge marking the final phase of the YD.

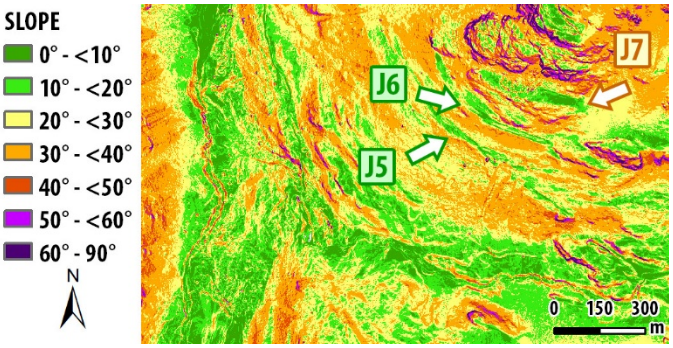

Figure S 3: Slope map of LG moraine set J7, J6 and J5. The landforms appear as linear elements in green-yellow color indicating lower slope angles, and are highlighted by arrows.

JAM-18-11 — ^{10}Be exposure age: 14,710 ± 270 yrs

COORDINATES N 46.8904 | E 10.1871
ALTITUDE 2520 m
L x B x H 2.7 x 1.3 x 1.1 m

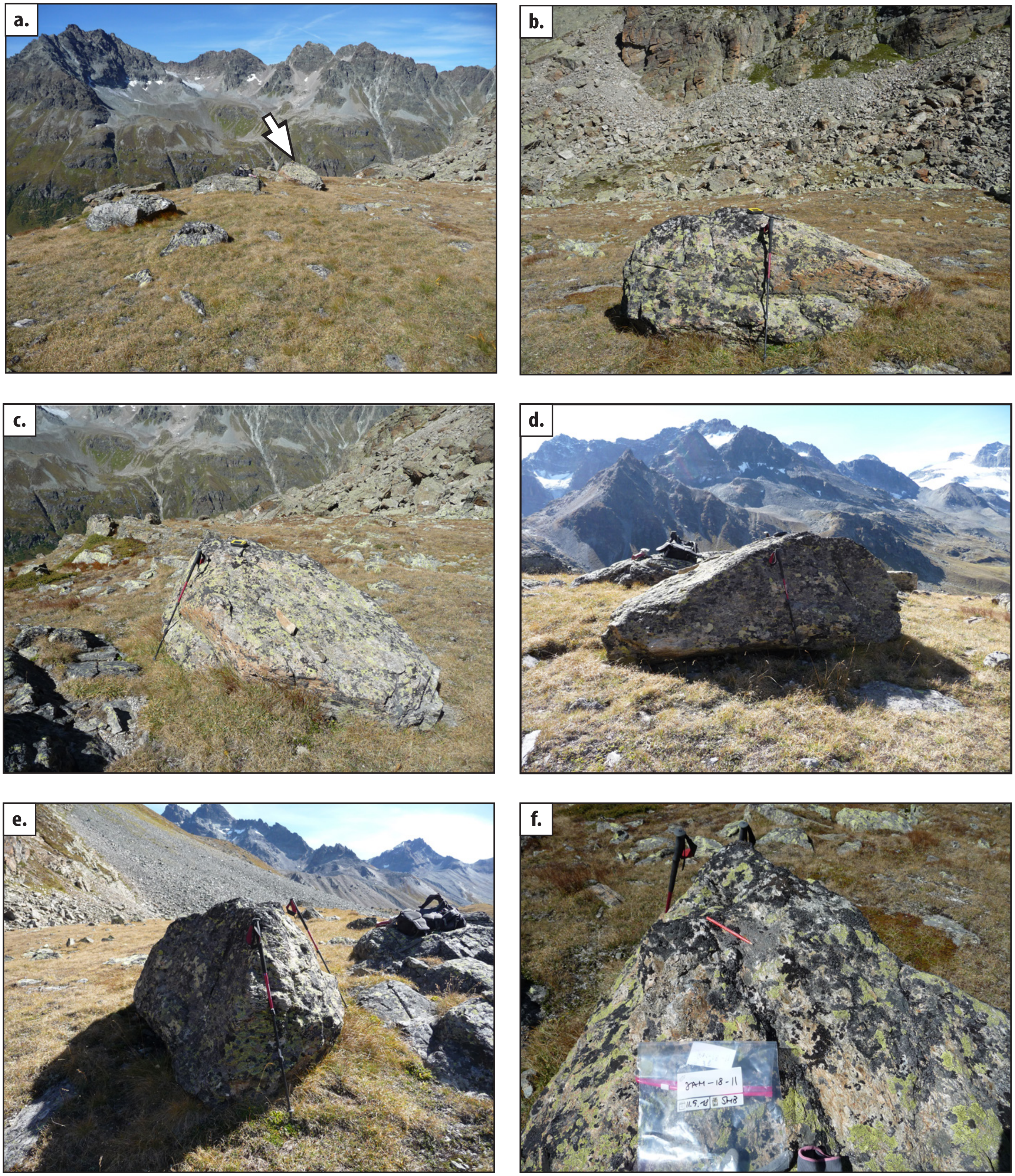

Figure S4: JAM-18-11. (a) View towards W. (b) View towards N. (c) View towards W. (d) View towards S with Augstenferner and Jamtalferner in the background. (e) View towards E. (f) Sampled rock surface indicated by pencil.

JAM-18-13 ^{10}Be exposure age: 12,880 ± 240 yrs

**COORDINATES**	**N 46.8901 | E 10.1838**
**ALTITUDE**	**2432 m**
**L x B x H**	**2.3 x 1.0 x 1.5 m**

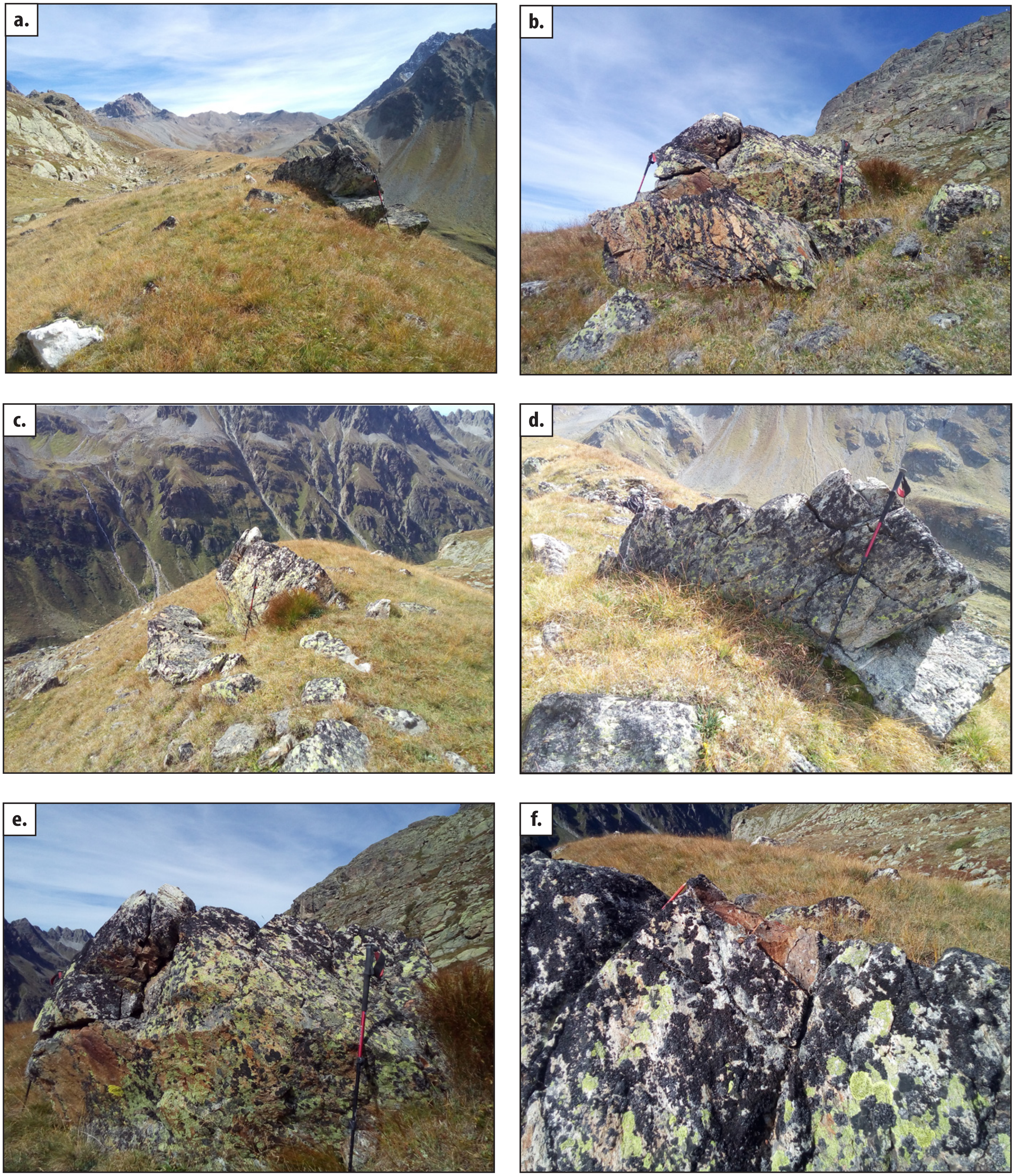

Figure S5: JAM-18-13. (a) View towards W. (b) View towards N. (c) View towards W. (d) View towards SE (Futschöltal). (e) View towards NW. (f) Sampled rock surface indicated by pencil.

JAM-19-19 — ^{10}Be exposure age: 14,420 ± 270 yrs

COORDINATES N 46.8904 | E 10.1875
ALTITUDE 2522 m
L x B x H 2.0 x 1.2 x 0.9m

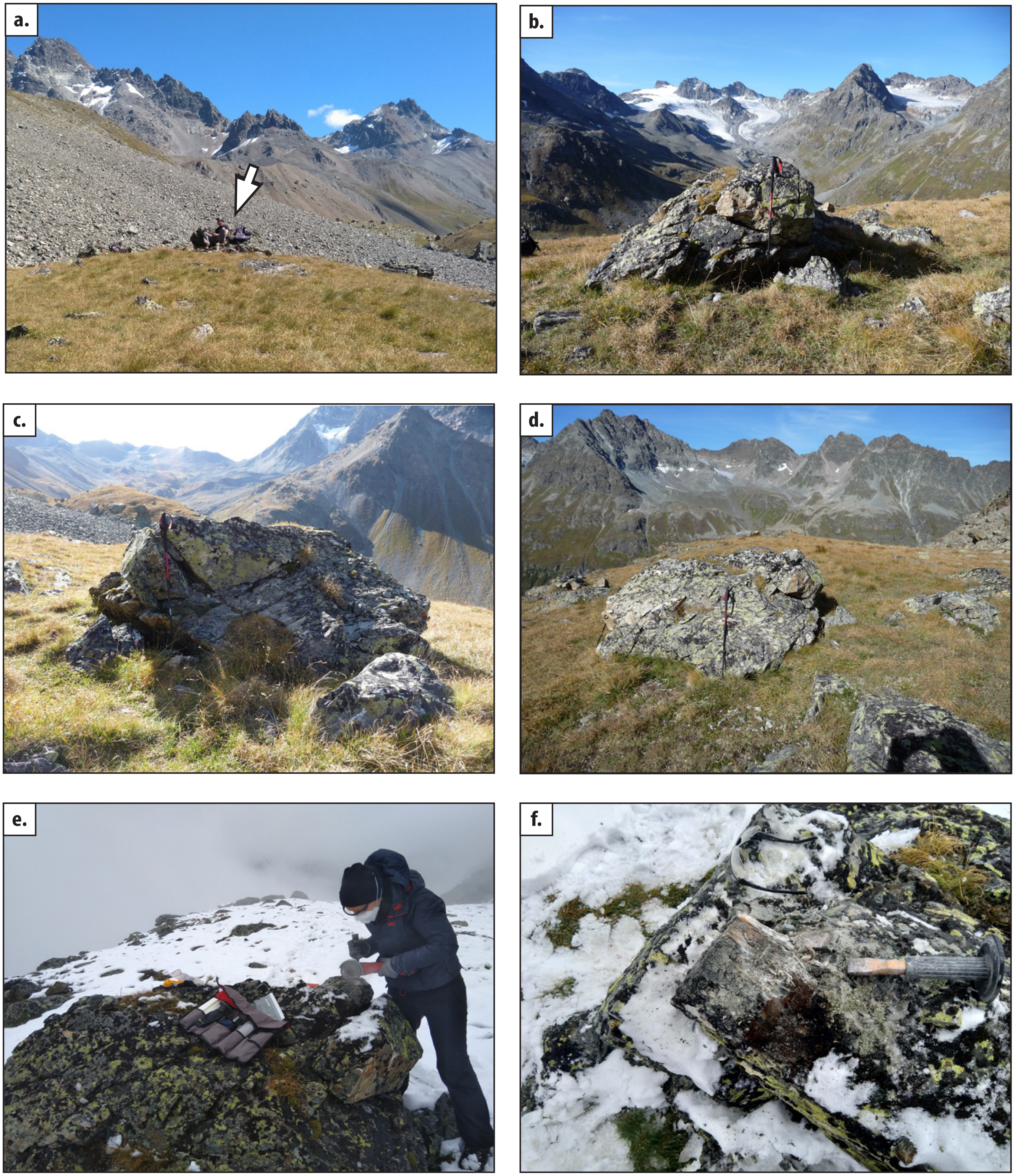

Figure S6: JAM-19-19. (a) View towards E; note person sitting on the boulder for scale. (b) View towards S with Jamtalferner and Totenfeld in the background. (c) View towards SE. (d) View towards W. (e) Sampling, view towards W. (f) Sampled rock surface.

JAM-19-20 — ^{10}Be exposure age: 12,120 ± 230 yrs

COORDINATES N 46.8893 | E 10.1865
ALTITUDE 2445 m
L x B x H 1.9 x 0.7 x 0.6 m

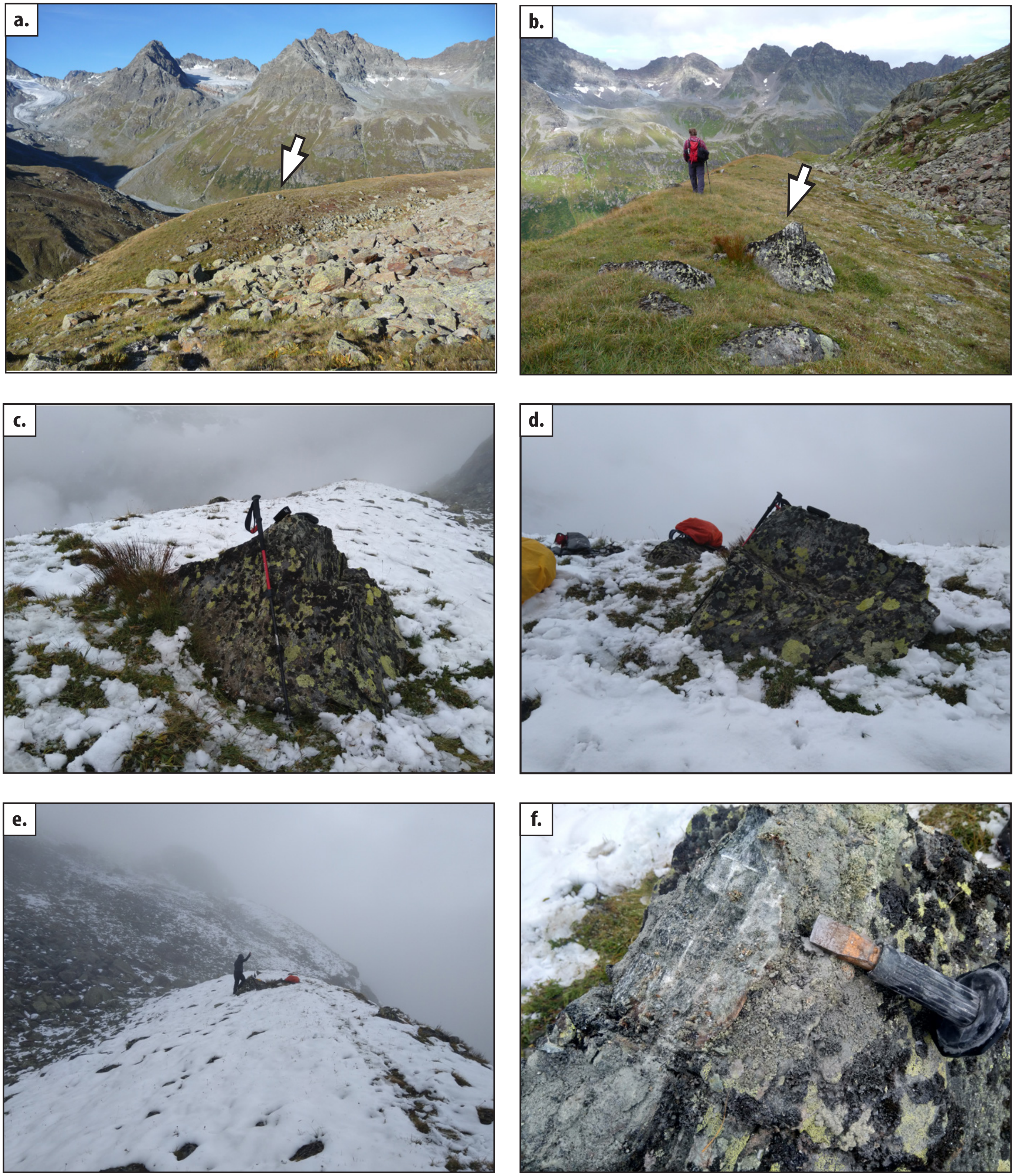

Figure S7: JAM-19-10. (a) View towards SW. (b) View towards W. (c) View towards W (close-up). (d) View towards S. (e) Sampling, view towards E. (f) Sampled rock surface.

JAM-20-23 — ^{10}Be exposure age: 14,130 ± 290 yrs

COORDINATES N 46.8904 | E 10.1872
ALTITUDE 2521 m
L x B x H 2.5 x 1.3 x 0.9 m

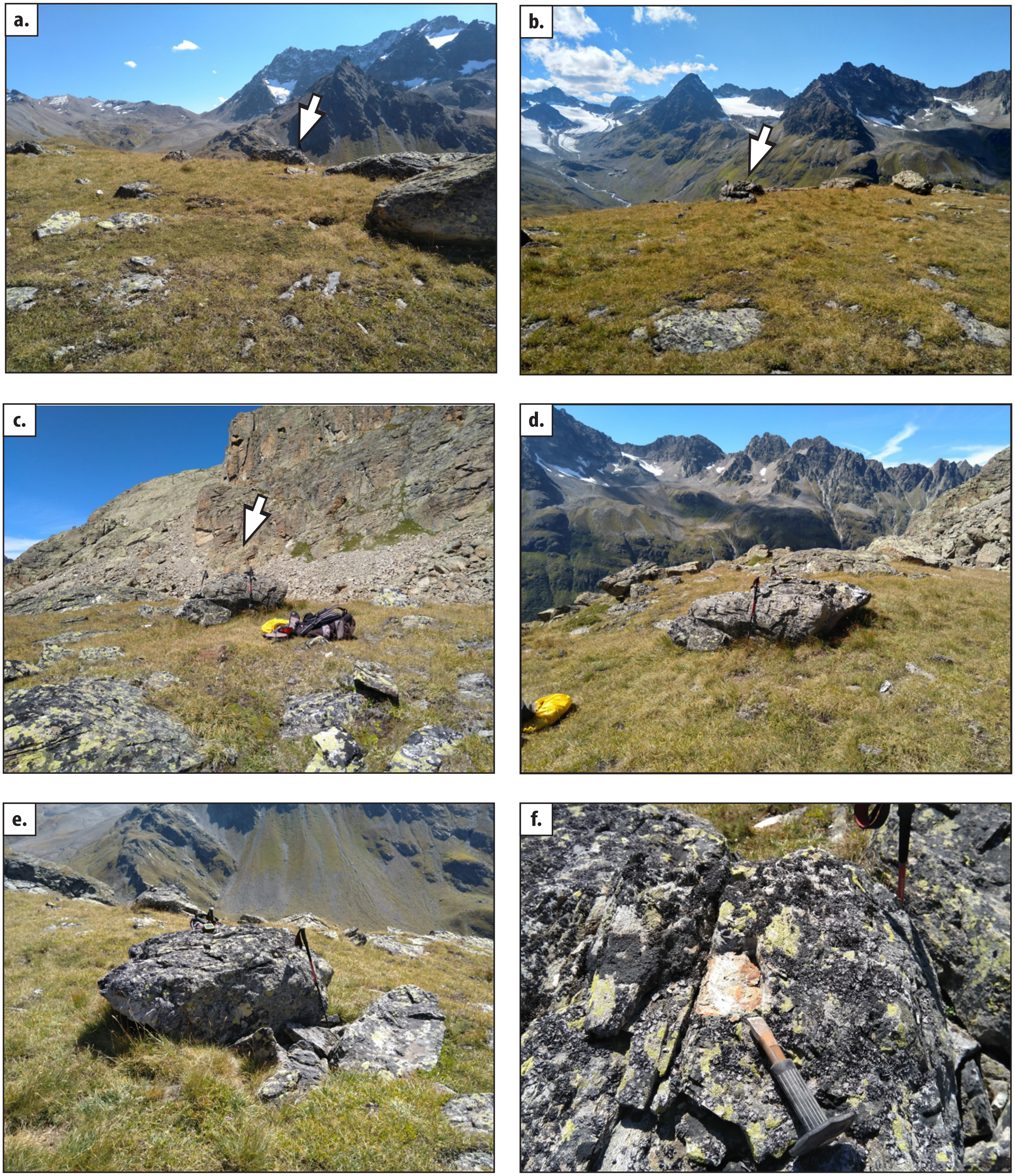

Figure S8: JAM-20-23. (a) View towards SE with Augustenferner and Futschölpass in the background. (b) View towards SSW with Totenfeld and Jamtalferner in the background. (c) View towards NNE. (d) View towards W. (e) View towards SSE. (f) Sampled rock surface.

JAM-20-24 — ^{10}Be exposure age: 11,620 ± 220 yrs

COORDINATES N 46.8895 | E 10.1829
ALTITUDE 2380 m
L x B x H 1.9 x 1.7 x 1.3 m

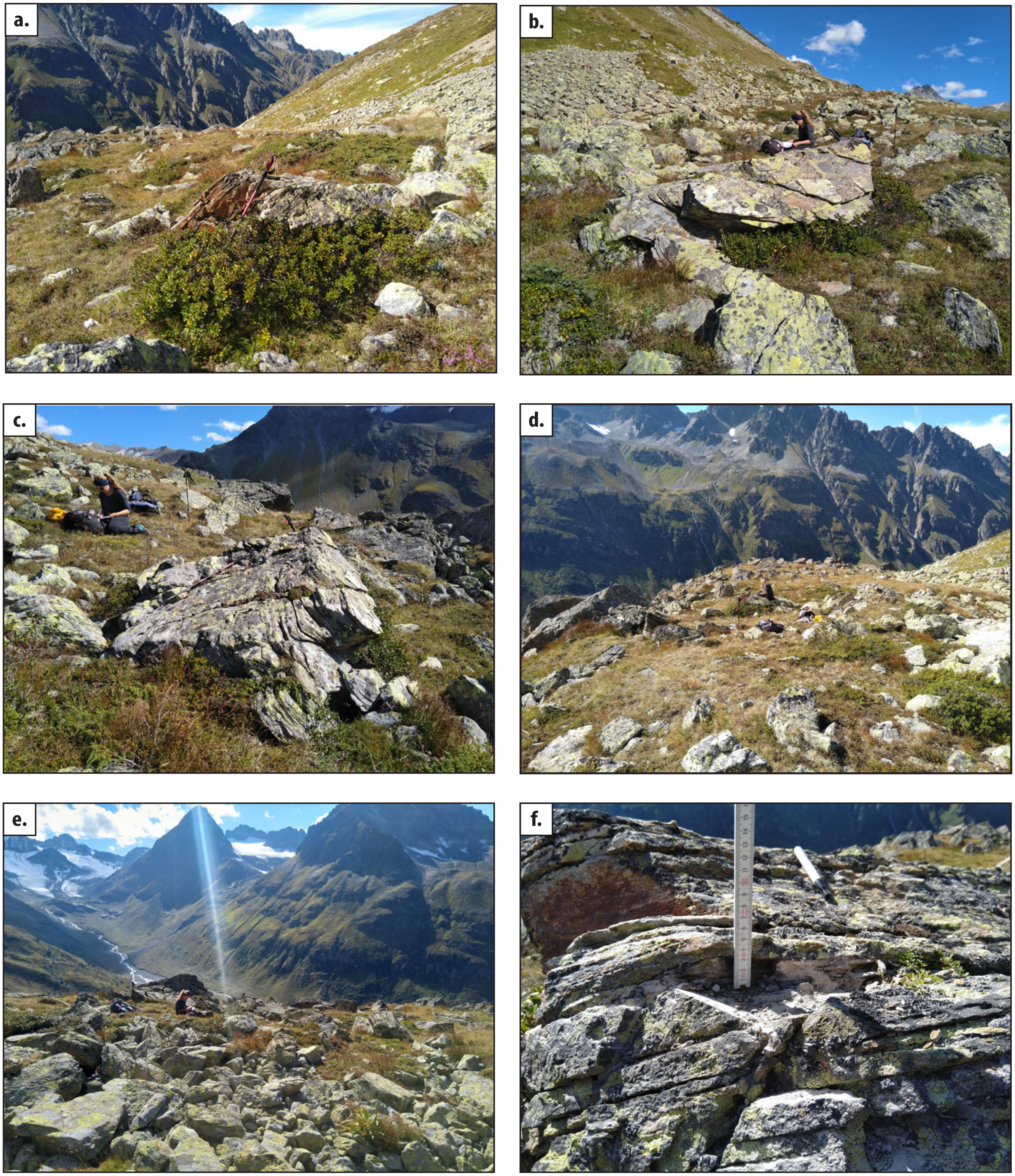

Figure S9: JAM-20-24. (a) View towards SW. (b) View towards E. (c) View towards SW. (d) View towards W. (e) View towards S. (f) Sampled rock surface.

COORDINATES N 46.8893 | E 10.1835
ALTITUDE 2389 m
L x B x H 2.0 x 1.5 x 1.1 m

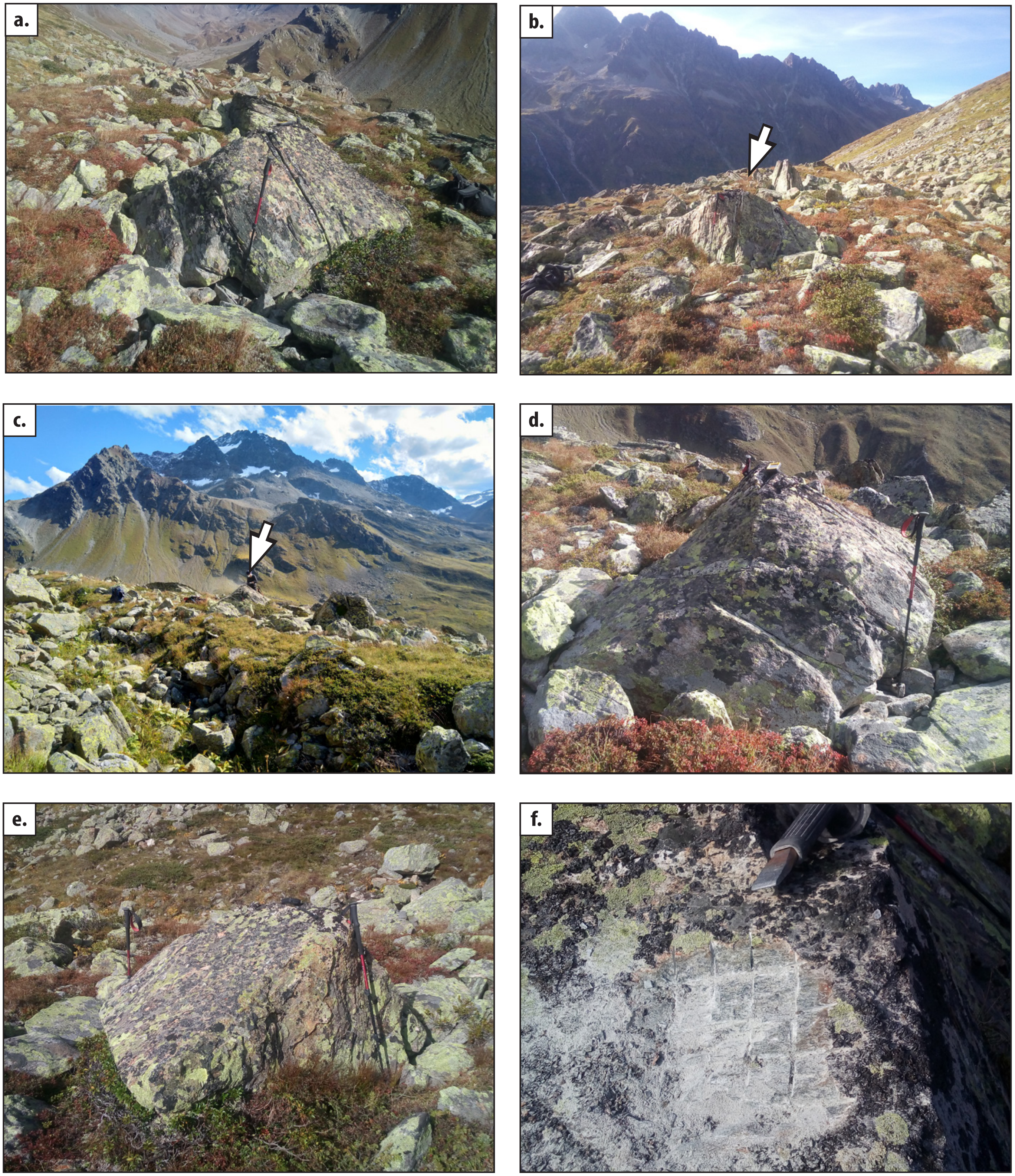

Figure S10: JAM-20-25. (a) View towards E. (b) View towards W. (c) View towards SW with Augustenferner in the background. (d) View towards S. (e) View towards N. (f) Sampled rock surface.

JAM-20-26 — ^{10}Be exposure age: 10,490 ± 220 yrs

COORDINATES N 46.8898 | E 10.1740
ALTITUDE 2065 m
L x B x H 2.3 x 1.9 x 0.5 m

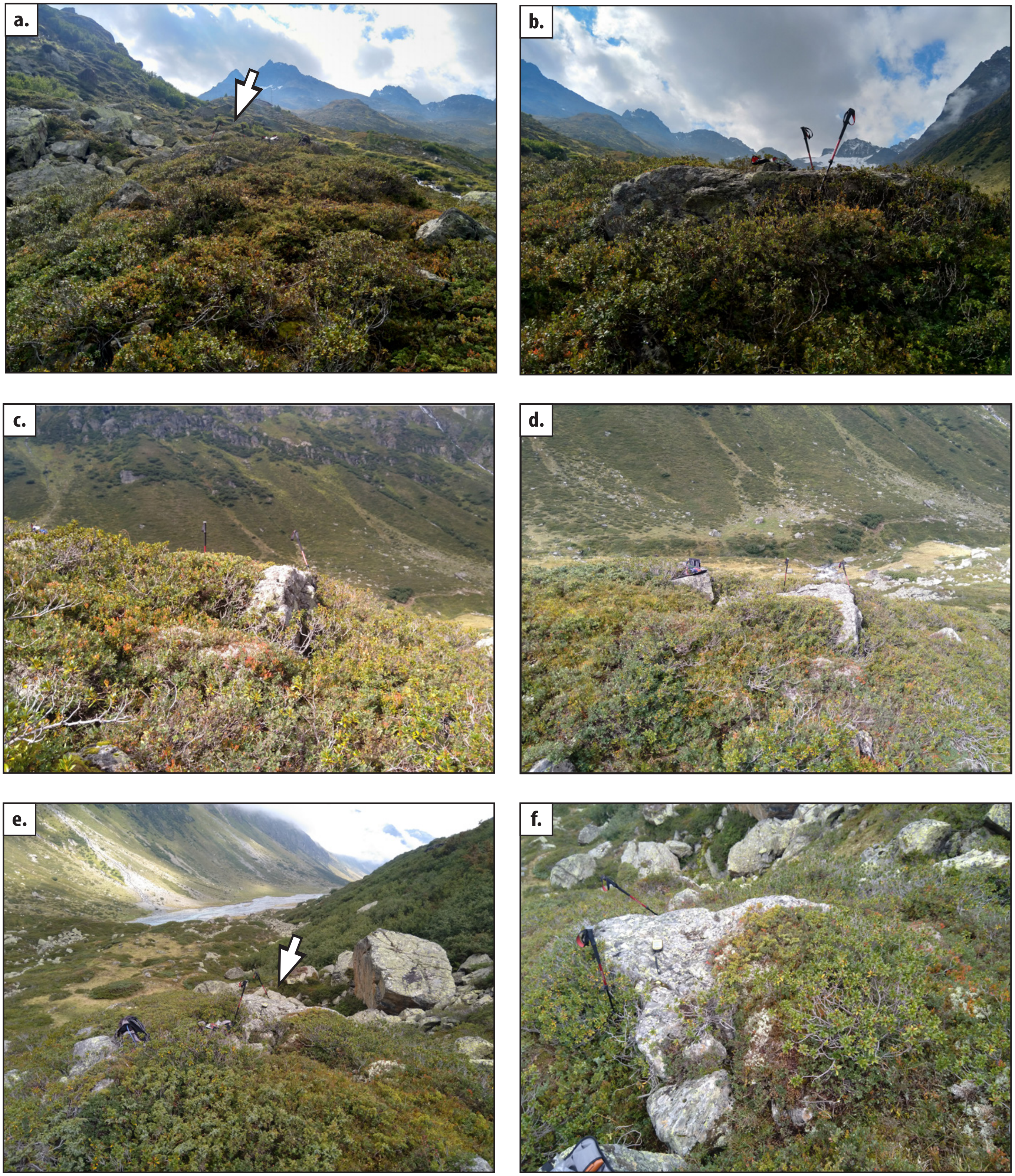

Figure S11: JAM-20-26. (a) View towards SE. (b) View towards S with Jamtalferner in the background. (c) View towards W. (d) View towards NW. (e) View towards N with Jambach (floodplane) in the background. (f) Sampled rock surface next to handheld GPS device.

JAM-20-27 — ^{10}Be exposure age: 11,650 ± 220 yrs

COORDINATES N 46.8908 | E 10.1733
ALTITUDE 2047 m
L x B x H 2.0 x 1.7 x 0.8 m

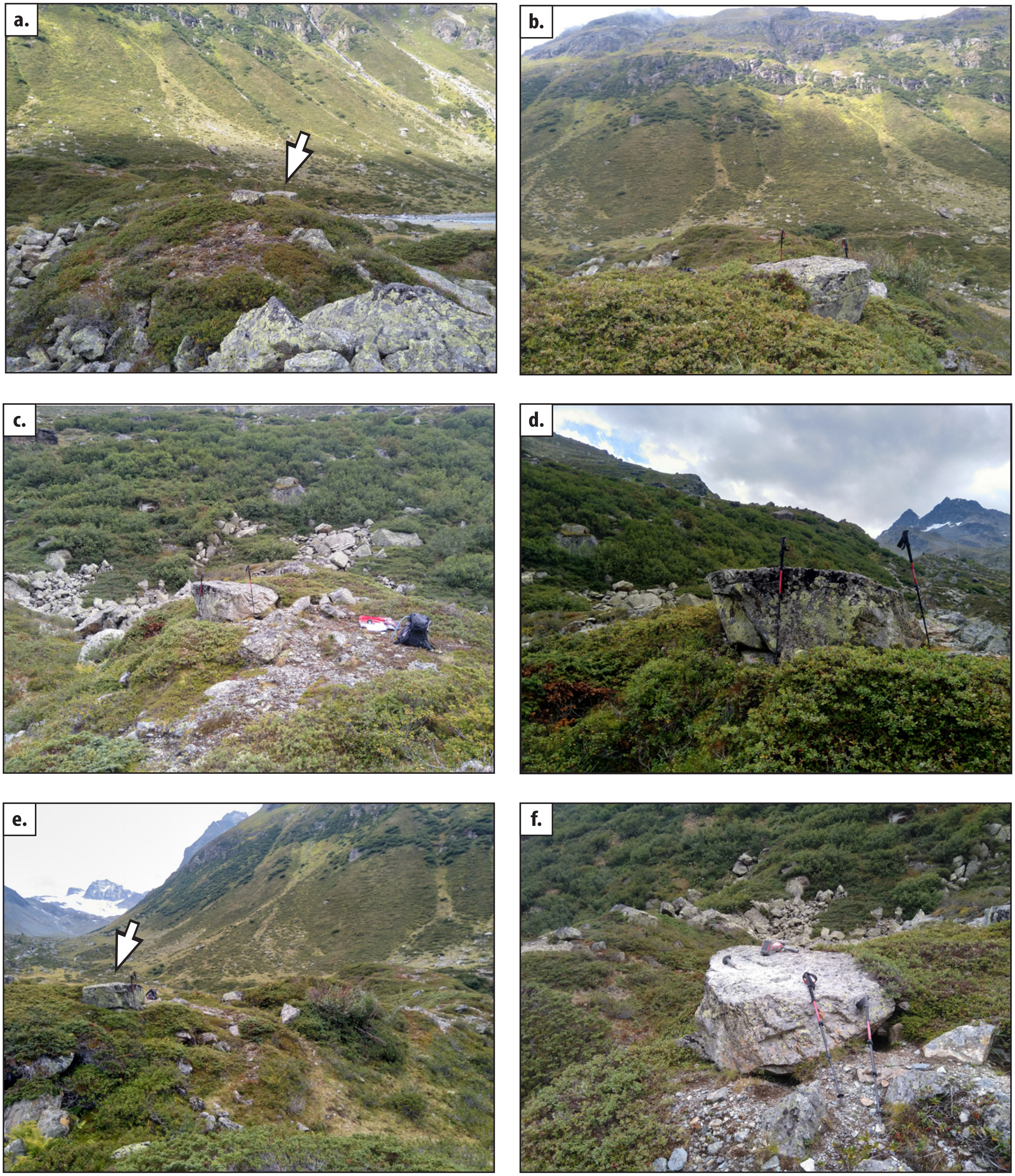

Figure S12: JAM-20-27. (a) View towards NW. (b) View towards W. (c) View towards E. (d) View towards SSE. (e) View towards SSE. (f) Sampled rock surface next to chisel.

FMB-18-04 — ^{10}Be exposure age: 11,460 ± 240 yrs

COORDINATES N 46.9429 | E 10.2697
ALTITUDE 2044 m
L x B x H 2.6 x 1.8 x 1.5 m

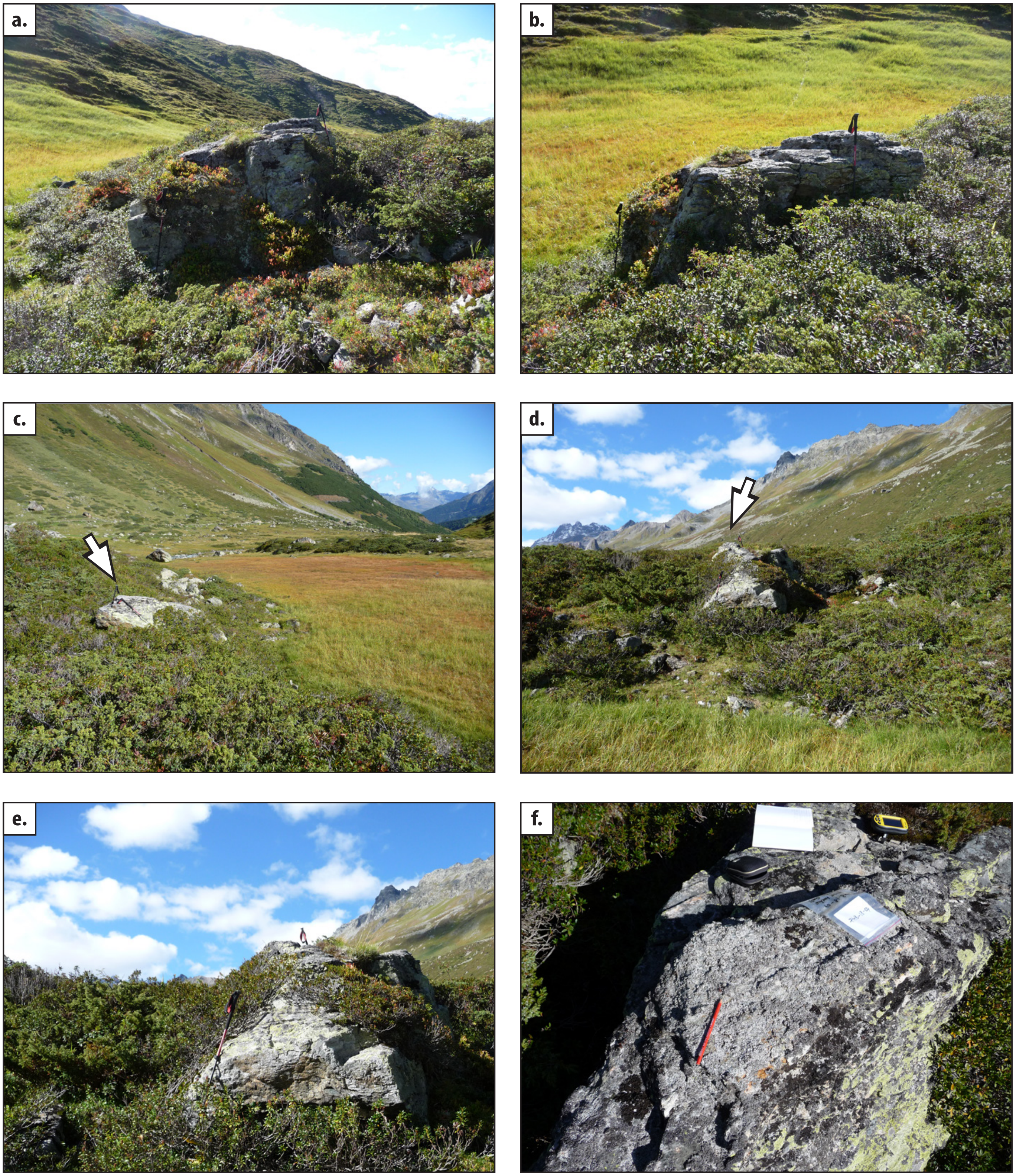

Figure S13: FMB-18-04. (a) View towards SE; note poles (length ca. 1.10 m) for scale. (b) View towards E. (c) View towards NW (downstream) with moraine F4 in the background. (d) View towards SSW. (e) View towards SSW (closeup). (f) Sampled rock surface indicated by pencil.

FMB-18-05 — ^{10}Be exposure age: 14,000 ± 280 yrs

**COORDINATES**	**N 46.9436 | E 10.2698**
**ALTITUDE**	**2040 m**
**L x B x H**	**6.0 x 3.0 x 1.9 m**

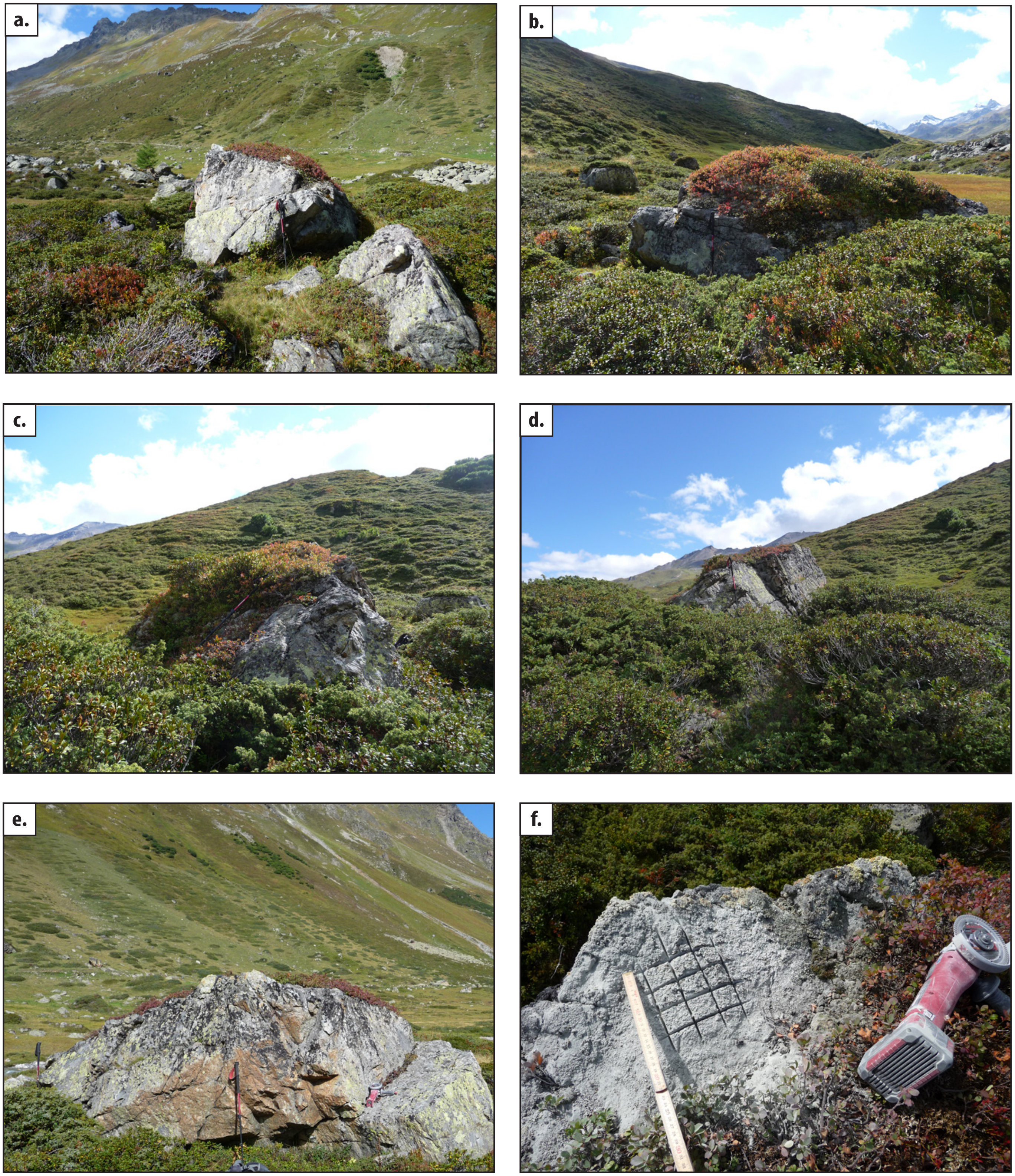

Figure S14: FMB-18-05. (a) View towards SW. (b) View towards S with F3 (middle-right section) in the background. (c) View towards E. (d) View towards NE. (e) View towards NW. (f) Sampled rock surface.

FMB-18-08 ^{10}Be exposure age: 11,920 ± 220 yrs

COORDINATES N 46.9438 | E 10.2688
ALTITUDE 2043 m
L x B x H 2.0 x 1.0 x 1.2 m

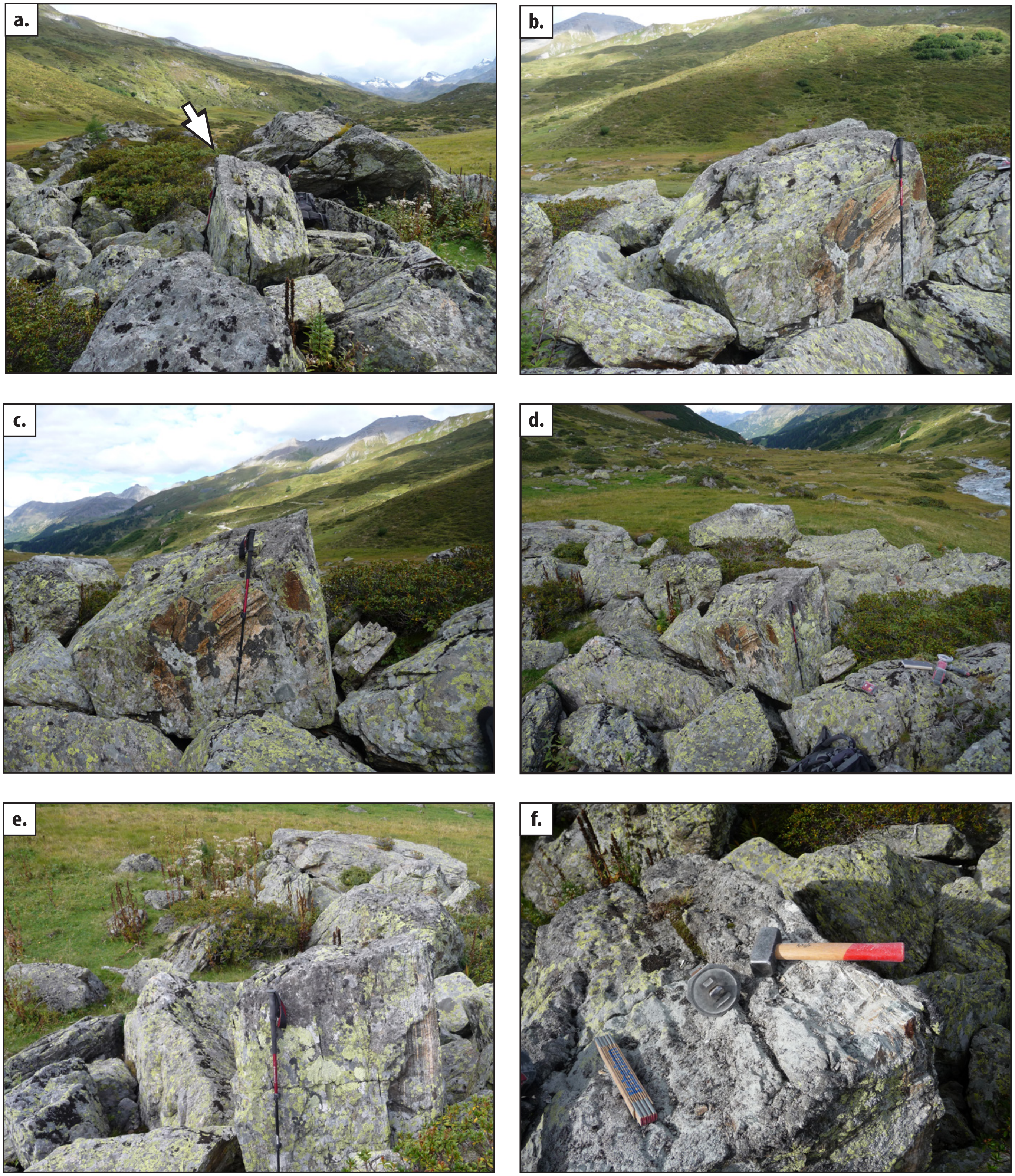

Figure S15: FMB-18-08. (a) View towards S. (b) View towards E. (c) View towards NE. (d) View towards N. (e) View towards W. (f) Sampled rock surface.

FMB-18-09 ^{10}Be exposure age: 9100 ± 170 yrs

**COORDINATES**	**N 46.9438 | E 10.2689**
**ALTITUDE**	**2042 m**
**L x B x H**	**1.1 x 1.5 x 1.4 m**

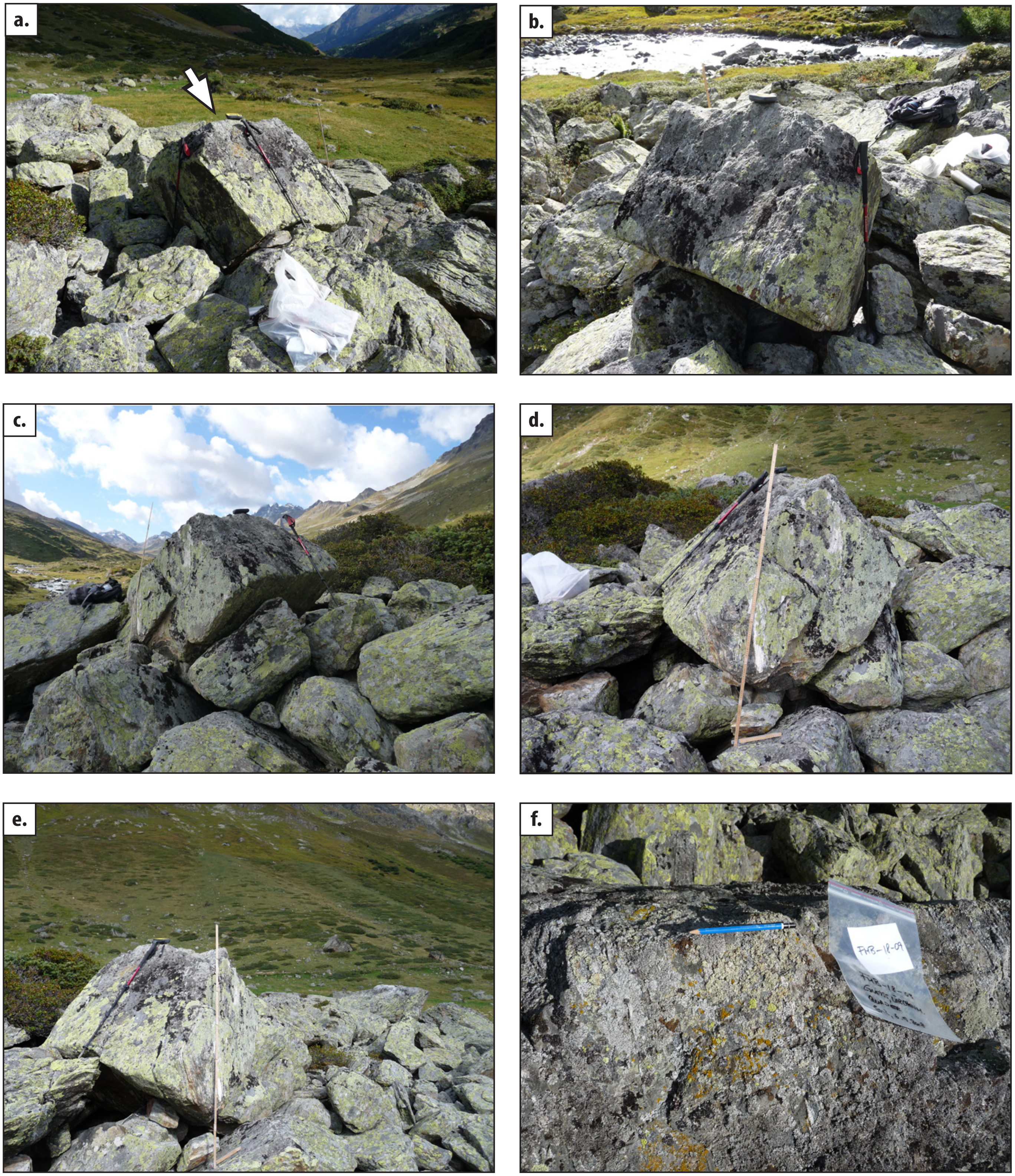

Figure S16: FMB-18-09. (a) View towards N. (b) View towards E with Fimbabach (creek) in the background. (c) View towards S. (d) View towards W. (e) View towards NW. (f) Sampled rock surface indicated by pencil.

FMB-19-12 ^{10}Be exposure age: 11,590 ± 220 yrs

COORDINATES N 46.9435 | E 10.2699
ALTITUDE 2041 m
L x B x H 2.5 x 2 .0 x 1.1 m

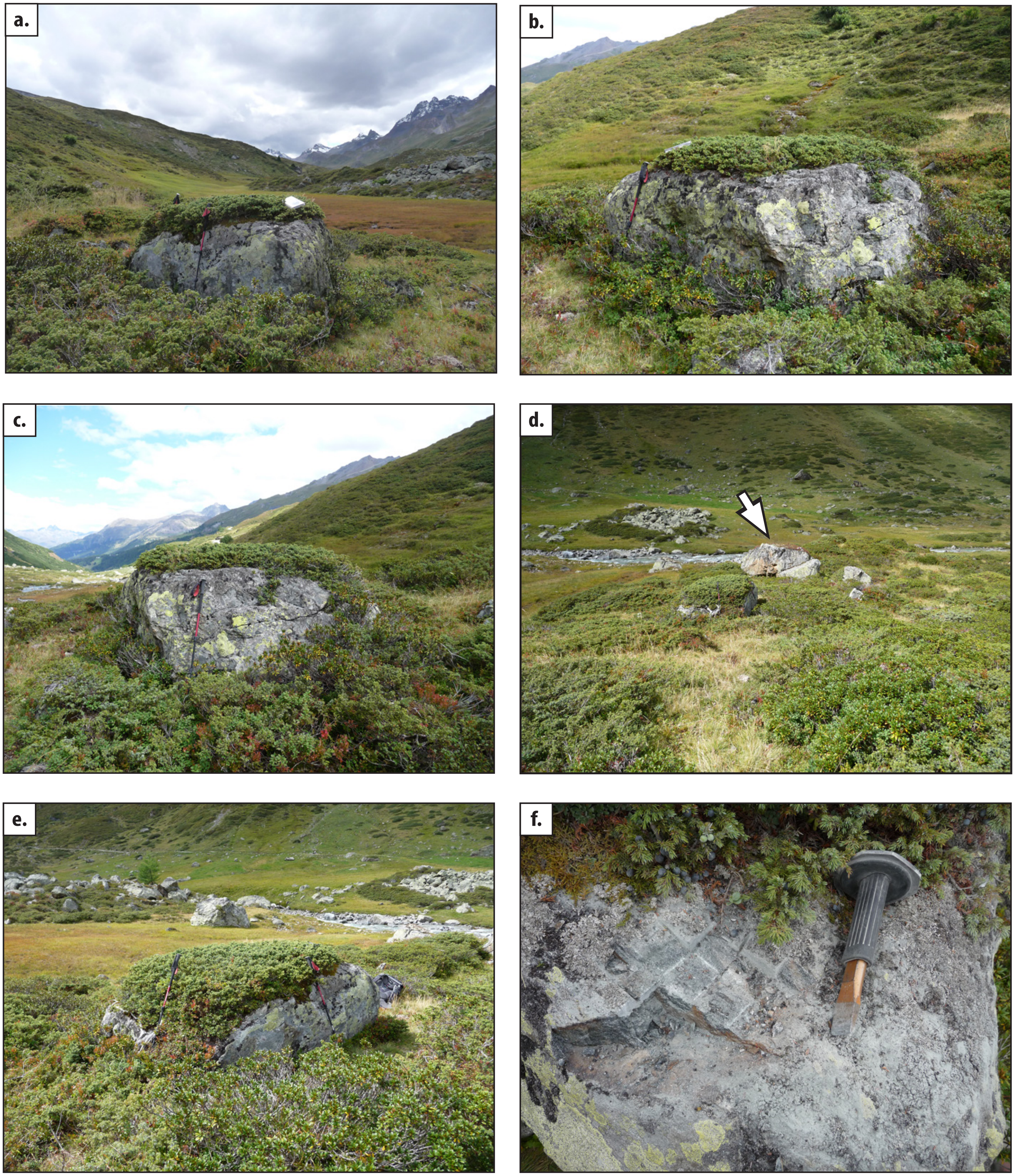

Figure S17: FMB-19-12. (a) View towards S. (b) View towards E. (c) View towards N. (d) View towards W. (e) View towards SW with F3 with little tree on top in the background. (f) Sampled rock surface.

FMB-19-13 — ^{10}Be exposure age: 10,760 ± 200 yrs

COORDINATES N 46.9432 | E 10.2694
ALTITUDE 2044 m
L x B x H 2.9 x 2.0 x 0.7 m

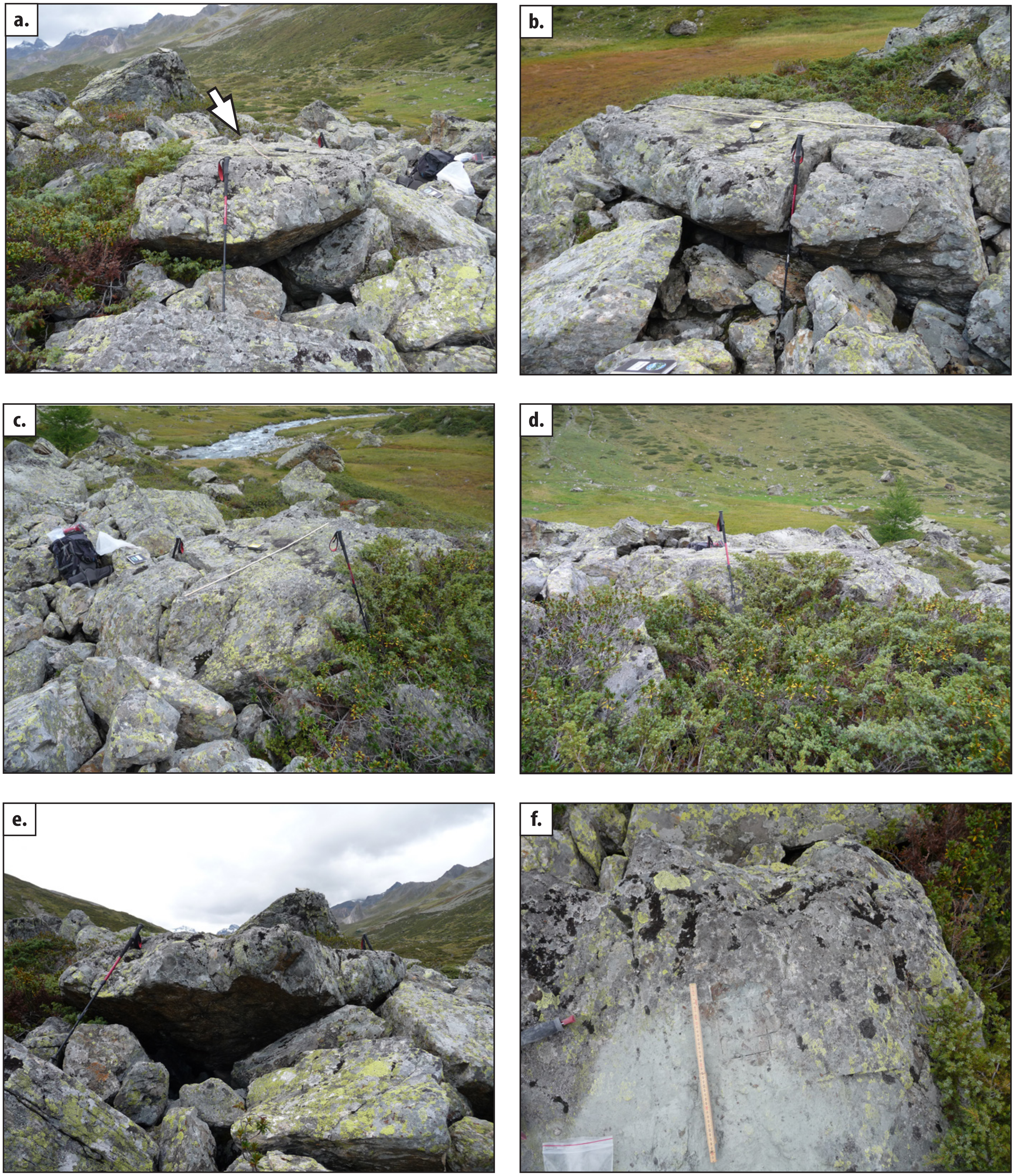

Figure S18: FMB-19-13. (a) View towards SW. (b) View towards E. (c) View towards N. (d) View towards W. (e) View towards S. (f) Sampled rock surface.

REFERENCES

1 *Schaefer, J. M. et al. High-Frequency Holocene Glacier Fluctuations in New Zealand Differ from the Northern Signature. Science 324, 622-625, doi:10.1126/science.1169312 (2009).*

2 *Braumann, S. M. et al. Holocene glacier change in the Silvretta Massif (Austrian Alps) constrained by a new Be-10 chronology, historical records and modern observations. Quaternary Sci Rev 245, doi:10.1016/j.quascirev.2020.106493 (2020).*
